# Evaluation of ceftriaxone pharmacokinetics in hospitalized Egyptian pediatric patients

**DOI:** 10.1007/s00431-023-05091-0

**Published:** 2023-07-24

**Authors:** Mohamed W. Eldougdoug, Doaa M. Youssef, Amal S. El-Shal, Yasmine Ahmed Sharaf, Sridivya Raparla, Bhaskara R. Jasti, Hanan M. Elnahas

**Affiliations:** 1 Department of Pharmacy Practice, Faculty of Pharmacy, Zagazig University, Zagazig, Egypt; 2 Department of Pediatrics, Faculty of Medicine, Zagazig University, Zagazig, Egypt; 3 Department of Medical Biochemistry and Molecular Biology, Faculty of Medicine, Zagazig University, Zagazig, Egypt; 4 Department of Medical Biochemistry and Molecular Biology, Armed Forces College of Medicine (AFCM), Cairo, Egypt; 5 Pharmaceutical Analytical Chemistry Department, Faculty of Pharmacy, Zagazig University, Zagazig, Egypt; 6 Department of Pharmaceutics & Medicinal Chemistry, Thomas J. Long School of Pharmacy and Health Sciences, University of the Pacific, Stockton, CA USA; 7 Department of Pharmaceutics & Industrial Pharmacy, Faculty of Pharmacy, Zagazig University, Zagazig, Egypt

**Keywords:** Pharmacokinetics, Ceftriaxone, Pediatric, HPLC, Hypoalbuminemia, Cholestasis

## Abstract

This study aimed to evaluate ceftriaxone pharmacokinetics that affects the achievement of targets in the treatment of critically ill children (meningitis, pneumonia, urinary tract infection, peritonitis, and infective endocarditis( who were admitted to Zagazig University Pediatric hospital in Egypt to monitor for the drug adverse effects.

Blood samples were obtained from 24 hospitalized pediatric patients (ages ranging from 2.5 months to 12 years) after administering the calculated dose of ceftriaxone via intravenous bolus route. Then, ceftriaxone plasma concentrations were measured using a validated HPLC method with ultraviolet detection. The pharmacokinetic analysis was conducted using Phoenix Winnonlin Program^®^ software.

Data for total and free ceftriaxone best fitted on a one-compartment model with the first-order elimination process. Clearance of ceftriaxone is reduced for patients with reduced kidney function and increased with those with augmented renal clearance. The volume of distribution and the free fraction are increased in these patients, especially those with hypoalbuminemia with a shorter half-life time were detected. A slight increase in total bilirubin and liver enzymes has been observed after treatment with ceftriaxone in these patients.

*  Conclusion*: In most critically ill pediatric patients, the current ceftriaxone treatment regimen (50 to 100 mg/kg) offers adequate pathogenic coverage. The clearance of free ceftriaxone in all patients correlates well with their renal function (eGFR), with r2 = 0.7252. During therapy with ceftriaxone at all doses ranging from 50 to 100 mg/kg, a rise in total bilirubin was observed in these patients. Moreover, liver enzymes (ALT and AST) increased moderately (*p* 0.0001). So, it is recommended to monitor total bilirubin and liver enzymes during the treatment with ceftriaxone, especially for a long duration (more than 5 days) or use another agent in patients with high baseline values.

**Table:** 

**What is Known:** *• The dosing regimen of ceftriaxone (50 to 100 mg/kg) provided optimum therapeutic outcomes.* *• Some studies show data for total and free Ceftriaxone best fitted on a one-compartment model while other studies show data for total and free Ceftriaxone best fitted on a two-compartment model.*	
**What is New:** *•* *Up to my knowledge this is the first study ,considering individual pharmacokinetic analysis, conducted on hospitalized Egyptian pediatric population most of them with reduced kidney function with ages ranging from 2.5 months to 12 years. Data for total and free Ceftriaxone best fitted on a one-compartment model with linear clearance of the free ceftriaxone.* *•* *In all patients, total bilirubin and liver function tests were mildly increased, making them at risk for cholestasis or ceftriaxone-induced cholestatic hepatitis.*

**Supplementary Information:**

The online version contains supplementary material available at 10.1007/s00431-023-05091-0.

## Introduction

Cephalosporins are a group of antibiotics that belong to β-lactams that were first isolated from the fungus *Acremonium Chrysogenum.* They exert their antibacterial activity via inhibition of the cell wall synthesis of the bacterial cell [1]. They bind to the penicillin-binding proteins (PBPs), essential in synthesizing bacterial cell wall peptidoglycans. As a result, cell lysis occurs in the hypo-osmotic or iso-osmotic environment surrounding the bacterial cell [2]. Cephalosporins are classified into five generations and are effective against aerobic and anaerobic microorganisms [3]. Ceftriaxone is a third-generation cephalosporin administered parenterally via intravenous or intramuscular injection. Ceftriaxone is commonly used in pediatrics and has indications for bloodstream infections caused by the following micro-organisms: *Streptococcus pneumoniae*, *Klebsiella pneumoniae*, *Staphylococcus aureus*, *Hemophilus influenzae* or *Escherichia coli*, septic arthritis, osteomyelitis, intra-abdominal infection, bacterial meningitis, lower respiratory tract infections (pneumonia), urinary tract infection (including cystitis and pyelonephritis) and acute otitis media [4]. Most adverse pharmacological effects associated with cephalosporin antibiotics in the pediatric population were brought on by inappropriate drug use [5]. Antibiotics can be categorized into time-dependent and concentration-dependent antibiotics based on their kill characteristics [6].

Ceftriaxone has time-dependent bacterial killing properties meaning that the maximum bacterial killing effect is achieved when the free plasma concentration of the drug remains above the minimum inhibitory concentration (*f*_T_ > MIC) for more than or equal to 60 to 70% of the dosing interval [7]. For most common infections, ceftriaxone’s MIC varies from 0.06 to 2 mg/L [5]. Also, according to the European Committee on antimicrobial susceptibility testing (EUCAST), the optimum MIC of ceftriaxone against most susceptible organisms ranges from 0.5 to 2 mg/L.

Ceftriaxone has an FDA block box warning for use in neonates (less or equal to 28 days), especially prematures, because of the risk of development of bilirubin encephalopathy. Also, mixing ceftriaxone with calcium containing IV solution (for example parenteral nutrition solutions) is contraindicated because of the risk of precipitation of ceftriaxone-calcium complex [8].

By shedding light on ceftriaxone pharmacokinetics, it has a high affinity for binding with serum albumin (85–95%). It has an elimination half-life (t_1/2_) of 9 h up to 30 days in neonates and 4 to 6.6 h in infants and children, which is a relatively long range. Ceftriaxone’s free fraction, not the bound fraction to plasma protein, is the only portion of the entire medication responsible for the antibacterial activity and the only fraction susceptible to elimination. This means that any condition (such as hepatic or renal disorders causing hypoalbuminemia or hyperbilirubinemia) leading to changes in the free fraction of the drug will modify the antibiotic exposure and have an impact on clinical outcomes. Ceftriaxone has mixed renal elimination (33–67% as an unaltered drug in urine) and biliary elimination (as an inactive drug in feces) [4].

The pharmacokinetic parameters of ceftriaxone’s free fraction are linear and dose-dependent, meaning that when the drug’s dose is increased, the drug’s steady-state concentration will increase accordingly [9].

Ceftriaxone dosage for children ranges from 50 to 100 mg per kg per day divided every 12 to 24 h for an average duration of five days (maybe less or more according to type and severity of infection) [4].

A previous pharmacokinetic study on pediatric patients with community-acquired pneumonia shows that the data best fit on a one-compartment model with first-order elimination kinetics, and the optimum dose that maintains the MIC for ceftriaxone at 2 mg/L was 100 mg/kg every 24 h [5].

Hoy WE …et al. documented in a systematic review the significant differences in antibiotic pharmacokinetics that may occur between different ethnic groups, so rational for this study was to be conducted on Egyptian pediatric patients to reveal data from such population [10]. Ethnicity has a great impact on the renal function. A large-scale study involving about 71,638 individuals from four ancestries for whom genome-wide association studies are carried out for estimated eGFR. Which identified different genes that affect the performance and sensitivity of renal cells in these different groups [11]. Furthermore, another published study by Fabian, June, et al. considering ethnicity’s effect on renal function supports this concept [12]. Thus, pharmacokinetics of ceftriaxone is affected.

This pharmacokinetic study was conducted to evaluate and shed light on the pharmacokinetics of ceftriaxone and a clinical evaluation of Egyptian pediatric patients in our setting. A validated HPLC method (with some modifications) with ultraviolet detection has been used to determine ceftriaxone plasma concentrations [9].

## Patients and methods

### Patients and clinical samples [9]

This cross-section observational study was conducted from May 2020 to November 2020. It involved 24 patients from different departments at the Zagazig University Pediatrics Hospital, Egypt, who fulfilled the following inclusion criteria: Egyptian with ages ranging from 1 month to < 18 years old and clinical indication for administration of ceftriaxone antibiotic. Patients who are non-Egyptian, with age < 1 month (neonates) or >  = 18 years old (adults), patients allergic to ceftriaxone, or patients with end-stage chronic kidney disease are excluded from this study. The ethics committee represented by the Institutional Review Board (IRB) at the Faculty of Medicine, Zagazig University, gave us approval for collecting blood samples from pediatric patients opportunistically during the duration of treatment (Approval Number; ZU-IRB#6070/26/4/2020). The sample was taken as a comprehensive sample due to the rare attendance of cases (4 cases or less/month), so the committee determined the sample to be 24 cases. It was previously calculated using *the online version of EPI info.* with the power of study of 80%.

The characteristics and laboratory data of the patients involved in this study are obtained from patients’ files at baseline and after completion of treatment with ceftriaxone (the decision of treatment with ceftriaxone was previously taken from the responsible staff for the selected patients). In addition, GFR was estimated for each patient according to the bedside Schwartz equation: [(0.413 × height (cm)) / Scr (mg/dL)] as recommended by the National Kidney Disease Education Program. Furthermore, serum creatinine values were determined using Jaffe Colorimetric method at Zagazig University Hospitals Central Laboratories. These collected data are presented in the "Results" section.

Then, ceftriaxone was administered in doses ranging from 50^1^ to 100 mg/kg via intravenous bolus injection to each patient. Then, blood samples were obtained from each patient during specific time previously determined by department administrator (the time preceding the next dose, trough), as one blood sample from different dosing intervals. Three blood samples were obtained from each patient, then stored immediately after collection at 2–8 °C in an icebox. After that, plasma was separated using centrifugation at 6000 rpm for 15 min and then analyzed using a suitable High-Performance Liquid Chromatographic method.

**Fig. 1:**
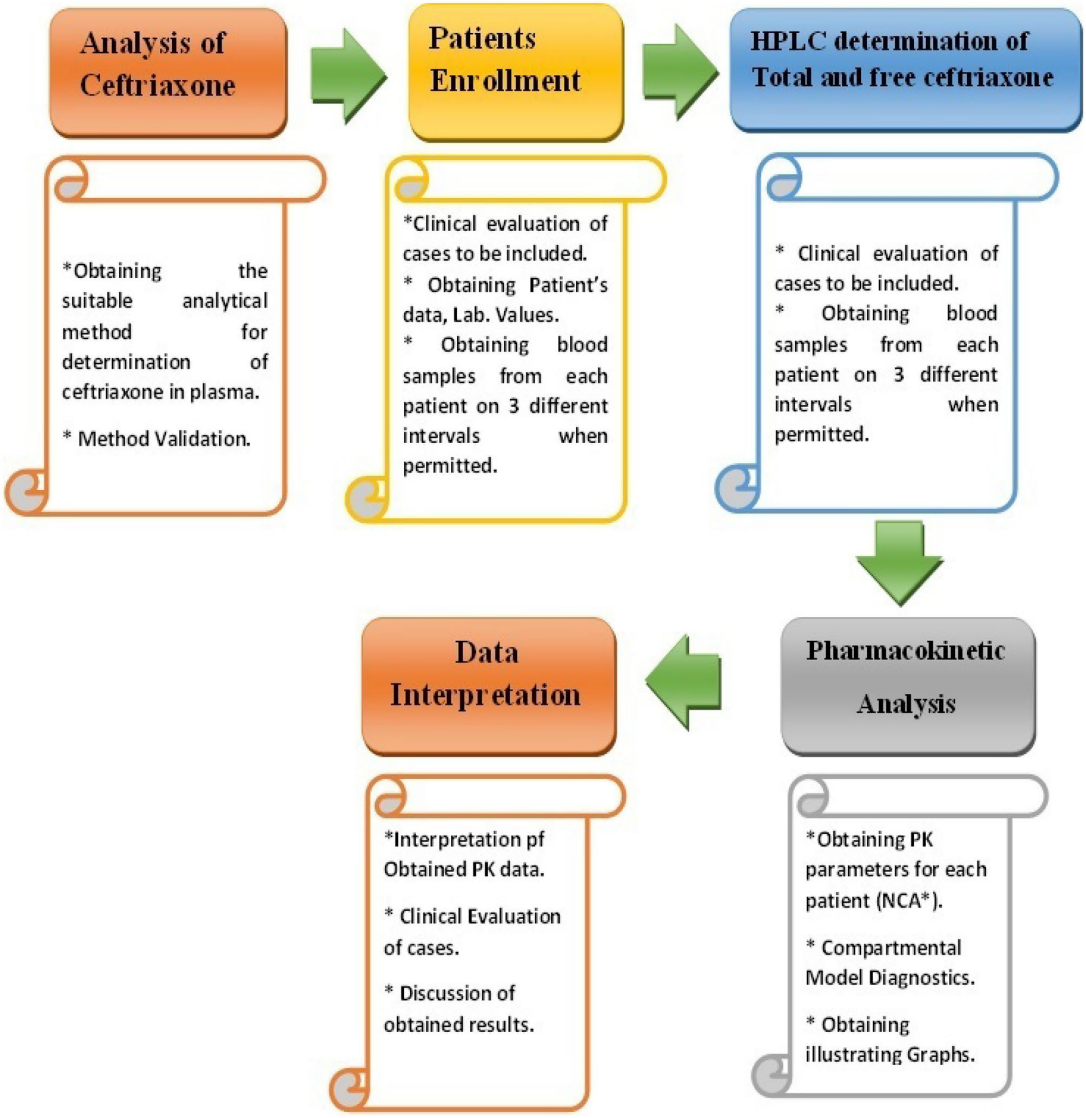
Study flow chart. *NCA; noncompartmental analysis. PK; pharmacokinetics

The analysis of each sample was carried out immediately after obtaining each blood sample. Both total and free ceftriaxone were determined. A study flow chart is illustrated by Fig. 1.

### Analysis of ceftriaxone

Blood samples were collected from patients (when available, 2 ml) in heparin tubes and then centrifuged at 6000 rpm for 15 min to obtain plasma. 250 μl of plasma is mixed with 250 μl of cold acetonitrile in an Eppendorf tube, then centrifuged at 8000 rpm for 6 min at 4 °C. The supernatant is then filtered and injected into the HPLC system for analysis [13].

Analysis was carried out as cited reference with some modifications using *Thermo Fisher Scientific*^*®*^*HPLC system*, which is composed of binary pumps, autosampler with a loop size of 10 μl, photodiode array detector (PDA) with detection at 260 nm, and *Chromquest 5.0 software* (*Thermo Electron Corp., Bellefonte, PA, USA*). Ceftriaxone analytical standard was kindly supplied from *EIPICO, Egypt.* Methanol, acetonitrile, and ammonium acetate (HPLC grades) were purchased from *Fisher Scientific, USA.* Double distilled water was applied all over the experiments and prepared in-house. A stock solution of ceftriaxone was freshly prepared by dissolving 20 mg of pure drug in 100 mL double distilled water to get a stock solution with a concentration of 200 µg mL^−1^ used for method validation.

Samples were analyzed by HPLC using *Agenla Technologies C18 column* (*250 mm* × *4.6 mm, 5 μm particle size*) maintained at 25 °C temperature. Elution pumps ran an isocratic flow using a mobile phase consisting of methanol and ammonium acetate 20 mM buffer solution (freshly prepared by dissolving 0.77 g in 500 ml distilled water) in the ratio of (79:21%v/v) at 1 ml/min flow rate. The autosampler utilizes water as a rinse solution, and the injection volume was 10 μl. The detection of samples was carried out at λmax 260 nm with a run time of 8 min [14].

### Pharmacokinetic analysis

Total and free ceftriaxone pharmacokinetics were analyzed separately with good fitting on a one-compartment model using *Phoenix Winnonlin Program*^*®*^(8.3.5.340, Core version 06-Feb. 2020). Then, the pharmacokinetic parameters were obtained and used to simulate the plasma concentration–time profile for each patient’s total and free ceftriaxone [9].

### Statistical analysis

*GraphPad Prism software* was used for statistical analysis of the changed laboratory values.

## Results

### HPLC method validation results

#### Construction of calibration curves in plasma

Aliquots of standard solutions, ranging from 2.5 to 100 μg per ml, were prepared in a series of 10 ml volumetric flasks, mixed with an equal volume of plasma from a healthy volunteer, then stirred. After sample pre-treatment, 10 μl of the supernatant was injected into the HPLC System. Detection was performed at wavelength 260 nm. The calibration graphs were constructed by plotting the peak areas obtained versus the corresponding injected concentrations (Fig. S1A).

HPLC chromatograms for method validation and examples for those from patients’ samples are presented in Figs. S1B–D, S7, and S8.

The applied Method was validated according to the ICH and FDA guidelines in respect to certain parameters such as linearity, precision, accuracy and system suitability [15, 16].

#### Data validation

Linearity was established across the specified range by plotting the area under the curve against its corresponding plasma concentration (Fig. S1, A). Linearity drug standards were injected in triplicates, and average responses were calculated. Table S2 shows linearity data, correlation coefficient, slope and intercept, and limits of detection (LOD) and quantification (LOQ) were calculated and listed. The result of LOD (3.3σ/S) was calculated as a function of the standard deviation of intercept (σ) and the slope (S) of the calibration curve, and LOQ was calculated as (10σ/S).

The method’s accuracy was confirmed using three standard concentrations injected in triplicates. The closeness of the calculated percentage recovery results to the actual values proved the method’s validity.

Precision was performed by three different determinations of three ceftriaxone concentrations on the same day (intra-day) and three other days (inter-day). Precision results listed in Table S2 show that the proposed method has good repeatability and reproducibility.

System suitability test parameters of the chromatographic method were checked and presented in table to ensure that the system was working correctly during the bioanalysis including retention time (Rt), column efficiency (number of theoretical plates (N), height equivalent to theoretical plates (HETP), tailing factor, resolution between ceftriaxone and plasma peaks (Rs) Table S2.

The influence of slight variations in one of the critical chromatographic parameters was evaluated while keeping all the others constant. The studied variables, including flow rate (1 ± 0.02) and methanol content (79% ± 1), were tested. Retention time and peak area were recorded in Table S3 upon these minor changes to indicate the robustness of the developed method.

### Clinical evaluation of patients

The patients in this study are 11 males and 13 females, with ages ranging from 2.5 months to 12 years old. Their demographics and the laboratory values at baseline and after therapy with ceftriaxone are listed in Table 1.

**Table 1:** Characteristics & laboratory data of 24 patients (baseline & after treatment values)

**Patient’s no.**	**Indication**	**Duration of** **treatment** **(days)**	**Sex**	**Age** **(years,** **months)**	**Weight** **(kg)**	**Height** **(cm)**	**Bilirubin** **(mg/dl)** **[T > up to 1.0]**			**Albumin** **(g/l)** **[3.5–5.5]**			**Serum creatinine** **( mg/dl)** **[0.6–1.4]**		**ALT** **(GPT)** **(IU/L)** **[Up to 40]**		**AST** **(GOT)** **(IU/L)** **[Up to 40]**		eGFR** (Schwartz Formula) **(ml/min/1.73 m**^**2**^**)**	
							B*		**A#**	**B**		**A**	**B**	**A**	**B**	**A**	**B**	**A**	**B**	**A**
**1**	Pneumonia	14	F	0 Y 9 M	9.5	70	0.2		0.28	3.7		3.9	1.1	0.5	10	20	27	35	**26.3**	**57.8**
**2**	Meningitis	10	F	0 Y 2.5 M	1.7	40	0.2		0.25	3.3		3.5	0.3	0.4	30	35	55	68	**55.1**	**41.3**
**3**	Urinary Tract Infection	5	F	3 Y 0 M	17.5	94	0.1		0.3	0.87		0.87	0.3	0.1	8.4	15	31.8	40	**129.4**	**388.2**
**4**	Peritonitis	14	M	9 Y 0 M	24	133.5	0.08		0.2	1.87		1.4	0.23	0.1	18.8	29	25.6	29	**239.7**	**551.4**
**5**	Infective Endocarditis	21	M	4 Y 0 M	14	103	0.3		0.4	4.35		3.74	1.96	1.06	4.1	13.8	9.4	37	**21.7**	**40.1**
**6**	Meningitis	10	M	12 Y 0 M	35	150	0.1		0.2	4.2		4.2	0.63	0.5	15	17	24	28	**98.3**	**123.9**
**7**	Pneumonia	14	F	1 Y 10 M	11	76	0.2		0.27	4.5		4.5	0.38	0.36	14	16	20	22	**82.6**	**87.2**
**8**	Meningitis	10	M	0 Y 8 M	9	70	0.1		0.2	4.1		4.1	0.5	0.42	15	20	50	60	**57.8**	**68.8**
**9**	Meningitis	14	M	0 Y 5 M	7.5	66	0.1		0.23	4		4	0.53	0.5	19	23	40	49	**51.4**	**54.5**
**10**	Pneumonia	7	F	2 Y 2 M	10.3	77	0.2		0.25	4.5		4.5	0.64	0.55	12	17	23	61	**49.7**	**57.8**
**11**	Meningitis	10	F	11 Y 0 M	34	145	0.2		0.29	4.4		4.4	1	0.9	16	18	25	29	**59.9**	**66.5**
**12**	Meningitis	10	F	1 Y 4 M	11.5	76	0.1		0.22	4.3		4.3	0.6	0.4	18	20	20	21	**52.3**	**78.5**
**13**	Gastroenteritis	7	M	1 Y 7 M	11	75	0.4		0.9	4.32		4.26	0.21	0.21	48.8	55	59.9	65	**147.5**	**147.5**
**14**	Meningitis, Gastroenteritis	10	M	0 Y 5 M	6.8	66	0.1		0.2	4		4.2	0.46	0.32	18	25	38	40	**59.3**	**85.2**
**15**	Gastroenteritis	7	F	0 Y 3 M	6.6	60	0.2		0.25	4.5		4.4	0.36	0.3	45.1	50	74	77	**68.8**	**82.6**
**16**	Meningitis	10	F	11 Y 0 M	15	154	0.2		0.27	4.35		4.3	0.64	0.5	51	54	75	79	**99.4**	**127.2**
**17**	Meningitis	7	M	0 Y 4 M	5.5	65	0.1		0.19	4.55		4.5	0.4	0.30	25	30	36	40	**67.1**	**89.5**
**18**	Urinary Tract Infection	5	F	5 Y 0 M	19	108	0.1		0.15	1.84		2	1.6	1.2	25	35	39	45	**27.9**	**37.2**
**19**	Pneumonia	14	F	0 Y 11 M	12	73	0.2		0.27	3.7		3.9	0.6	0.5	15	20	29	35	**50.2**	**60.3**
**20**	Meningitis	10	F	1 Y 0 M	13	78	0.28		0.35	3.2		3.3	0.5	0.4	39	45	49	69	**64.4**	**80.5**
**21**	Pneumonia	14	F	4 Y 0 M	16	107	0.2		0.4	4		4.2	1.85	1.4	25	29	45	50	**23.9**	**31.6**
**22**	Cystitis	7	M	10 Y 0 M	37	158	0.29		0.5	3		3.2	0.72	0.65	27	30	39	45	**90.6**	**100.4**
**23**	Gastroenteritis	7	M	0 Y 6 M	8	66	0.2		0.4	3.8		3.7	0.35	0.3	42	46	53	69	**77.9**	**90.9**
**24**	Pneumonia	14	M	6 Y 5 M	22	116	0.2		0.27	3.78		3.9	0.8	0.7	19	25	38	45	**59.9**	**68.4**
**Median**		**10**		**1.705**	**11.75**	**76.5**	**0.2**	**0.27**		**4**	**4.05**		**0.565**	**0.46**	**18.9**	**25**	**38**	**45**	**59.9**	**79.5**
**Minimum**		**5**		**0.21**	**1.7**	**40**	**0.08**	**0.15**		**0.87**	**0.87**		**0.21**	**0.1**	**4.1**	**13.8**	**9.4**	**21**	**21.7**	**31.6**
**Maximum**		**21**		**12**	**37**	**158**	**0.4**	**0.9**		**4.55**	**4.5**		**1.96**	**1.4**	**51**	**55**	**75**	**79**	**239.7**	**551.4**

*B baseline value; #A is the value after treatment with ceftriaxone

** According to National Kidney Foundation (NKF)*,* eGFR of 90 or higher is in the normal range, 60–89 may mean early kidney disease, 15–59 may indicate kidney disease and below 15 may mean kidney failure

*ALT* alanine aminotransferase, *AST* aspartate aminotransferase, *eGFR* estimated glomerular filtration rate. *Y *year, *M* month

Nine patients administer ceftriaxone because of meningitis (37.5%), six patients because of pneumonia (25%), three patients because of urinary tract infection (12.5%), one patient because of meningitis and gastroenteritis (≈ 4.167%), one patient because of peritonitis (≈ 4.167%) and one patient because of infective endocarditis (≈ 4.167%). Other associated medical conditions and the observed drug-drug interactions are recorded in Table S4.

The duration of treatment with ceftriaxone ranges from 5 to 21 days, with a median of 10 days. Only one patient was treated with ceftriaxone for more than 14 days, while the others received the drug for a duration equal to or less than 14 days.

All patients had a significant difference in total bilirubin values at baseline and after treatment, although within the normal range with a *p*-value of < 0.0001 and 95% CI from − 0.1607 to − 0.08017 and *r* of 0.8349. Also, liver enzymes, including ALT and AST, are mildly elevated with *p*-values of < 0.0001 and 95% CI values from − 6.409 to − 4.225 and from − 12.55 to − 5.144 and *r* of 0.9802 and 0.8678, respectively (Figure S2) (Table S1).

### Pharmacokinetics

In the case of total ceftriaxone concentration, none of the observed values were below the limit of quantification (BLOQ). All the observed concentration values for the total ceftriaxone were found to be within the lower limit of quantification (LLOQ) and did not require any method for imputation of BLOQ data.

In the case of free ceftriaxone concentration, observed data related to two subjects exhibited BLOQ values. These BLOQ values were replaced with half the value of LLOQ as per Beal’s M5 method for imputation of BLOQ data (Tables 2 and 3).

**Table 2:** Pharmacokinetic parameters of total and free ceftriaxone in 24 pediatric patients

**Patient #**	**Total Ceftriaxone**				**Free Ceftriaxone**				Unbound fraction average* (Observed range)**
	**Vss (ml)**	**CL (ml/h)**	**CL/eGFR**	**t**_**1/2**_** (h)**	**Vss (ml)**	**CL (ml/h)**	**CL/eGFR**	**t**_**1/2**_** (h)**	
1	8363.421	412.4198	1.051502	12.94588	35,460.42	1914.46	4.881092	13.38733	**21.54% (18.89–19.88%)**
2	1396.961	307.3588	1.174001	4.529875	2126.281	837.1903	3.197768	3.405622	**36.71% (25.01–26.3%)**
3	3664.44	737.8105	0.243197	5.430758	1042.449	1321.007	0.435431	4.495601	**55.85% (9.51–9.93%)**
4	4993.113	518.4681	0.065996	6.803175	9854.066	1315.172	0.167408	5.970423	**39.42% (34.9–35.4%)**
5	1101.492	152.4519	0.32001	7.972266	2531.102	539.0499	1.131515	5.202838	**28.28% (23.24–24.83%)**
6	9747.996	1808.401	0.439106	5.165824	3560.419	3864.769	0.938421	2.442022	**46.79% (12.83–14.48%)**
7	4851.421	1188.162	0.860485	3.520011	23,772.12	5026.279	3.640109	3.195727	**23.64% (28.11–29.33%)**
8	11,609.46	335.3665	0.400231	24.01689	27,315.06	1429.916	1.70648	15.23162	**23.45% (23.68–25.4%)**
9	657.1404	178.8306	0.270396	6.232549	823.856	406.6213	0.61482	5.8837	**43.98% (24.89–25.3%)**
10	4080.832	1375.985	1.702077	4.722829	18,475.9	6385.691	7.899025	3.757543	**21.55% (22.64–23.68%)**
11	1142.602	1040.619	0.428128	3.161246	20,037.39	10,895.35	4.482532	2.580582	**9.55% (17.49–19.1%)**
12	3638.064	125.8034	0.140682	21.21516	14,504.74	543.3068	0.607563	19.75682	**23.16% (23.48–24.95%)**
13	7049.659	475.8978	0.194214	17.06646	10,415.33	983.5143	0.401373	12.03742	**48.39% (51.49–51.95%)**
14	13,803.22	381.1512	0.525004	25.04822	25,617.56	1116.232	1.537518	17.82665	**34.15% (34.27–35.07%)**
15	11,630.04	439.7829	0.555145	17.6031	17,381.07	965.3229	1.218543	14.51582	**45.56% (38.31–39.68%)**
16	2101.914	227.3098	0.082318	12.82132	6293.145	1044.99	0.378432	7.4135	**21.75% (23.52–24.5%)**
17	8900.945	407.4808	0.555864	16.46693	27,100.14	1752.388	2.390518	10.41533	**23.25% (27.42–28.11%)**
18	10,352.60	272.33	0.372767	29.8707	23,989.37	1398.963	1.914917	15.91113	**19.47% (20.58–20.58%)**
19	198.2405	82.17014	0.095732	7.449968	93.55431	122.2342	0.142409	5.381362	**67.22% (25.84–26.79%)**
20	10,146.69	506.2888	0.426887	16.59243	29,930.87	2137.773	1.802503	10.72628	**23.68% (26.53–27.07%)**
21	8762.807	341.2257	0.596609	24.19684	5174.05	1088.907	1.903875	5.07728	**31.34% (27.18–28.28%)**
22	14,234.19	477.2572	0.11922	23.16377	16,007.16	1407.156	0.351511	9.399338	**33.92% (49.49–51.55%)**
23	10,679.26	393.1059	0.379899	20.70719	7398.431	1540.032	1.488293	6.157091	**25.53% (13.96–16.56%)**
24	17,133.47	468.1962	0.26766	26.16191	38,582.27	1194.369	0.682801	23.34325	**39.20% (42–42.14%)**
Median	**7706.54**	**409.9503**	**0.390065**	**14.7064**	**15,255.95**	**1318.089**	**1.353418**	**6.785295**	**30% (25.35%)**
Minimum	**198.2405**	**82.17014**	**0.065996**	**3.161246**	**93.55431**	**122.2342**	**0.142409**	**2.442022**	**9.6% (9.51%)**
Maximum	**17,133.47**	**1808.401**	**1.702077**	**29.8707**	**38,582.27**	**10,895.35**	**7.899025**	**23.34325**	**67.2% (51.95%)**

*Average Unbound fraction was calculated by dividing Cl of total ceftriaxone and Cl of free Ceftriaxone. [9] (Not necessary to fall within the range

** Observed Unbound fraction range from each patient [ (Concentration free/concentration total) × 100%]

*Vss* The volume of distribution at steady state, *CL* clearance, *eGFR* estimated glomerular filtration rate, *t1/2* half-life time

**Table 3:** Exposure parameters for total and free ceftriaxone in 24 patients

**Pt #**	**Total Ceftriaxone**				**Free Ceftriaxone**			
	**Cmax**	**C0**	**AUCall**	**AUMClast**	**Cmax**	**C0**	**AUCall**	**AUMClast**
	**ug/mL**	**ug/mL**	hr*ug/mL	hr*hr*ug/mL	**ug/mL**	**ug/mL**	hr*ug/mL	hr*hr*ug/mL
**1**	48.62	78.60	2195.50	33,886.29	9.30	20.09	475.20	6501.92
**2**	23.38	108.72	682.19	1396.23	6.15	47.52	273.95	365.82
**3**	12.02	84.14	1069.15	3165.16	1.19	56.60	636.83	312.65
**4**	70.11	155.34	1307.15	5013.23	24.82	74.53	569.96	1771.92
**5**	52.85	309.25	4057.60	14,482.55	13.12	95.92	1217.57	3585.60
**6**	27.60	117.53	782.84	1749.28	3.99	79.58	441.60	251.04
**7**	18.42	96.26	856.16	2312.23	5.33	21.42	201.10	669.13
**8**	49.28	72.45	1160.48	9649.98	12.52	31.85	400.60	2418.27
**9**	33.45	355.86	3924.73	7337.57	8.46	168.81	1780.62	1854.24
**10**	4.03	47.65	572.22	1060.30	0.95	10.25	124.13	249.79
**11**	14.44	284.12	1577.31	902.96	2.66	25.18	147.99	166.51
**12**	286.71	289.92	4921.79	38,132.49	71.55	73.46	1194.11	8988.98
**13**	22.39	118.51	1560.98	5663.87	11.63	62.44	820.48	2941.69
**14**	14.49	22.33	396.49	3491.49	5.08	12.31	183.69	1220.56
**15**	14.66	23.11	406.18	3528.58	5.82	16.18	231.05	1396.52
**16**	33.45	234.64	2713.16	7350.19	8.18	54.84	638.01	1795.96
**17**	23.78	54.69	807.99	5233.32	6.68	14.66	219.92	1469.35
**18**	45.79	108.60	1743.40	12,095.41	9.62	35.29	503.36	2538.98
**19**	59.33	1210.36	14,022.68	15,618.27	15.89	865.38	9708.74	4176.34
**20**	38.32	111.63	1677.75	9905.46	10.37	30.35	455.50	2680.00
**21**	33.76	125.50	1768.60	8541.37	9.55	67.57	853.00	2414.40
**22**	76.15	179.13	1414.54	5014.85	39.26	109.92	820.87	2578.32
**23**	20.08	45.28	708.67	5050.31	3.32	25.49	301.30	826.37
**24**	73.83	116.35	1023.40	4452.69	31.11	52.38	447.94	1875.45
**Median**	**33.45**	**113.99**	**1360.84**	**5141.82**	**8.88**	**49.95**	**465.35**	**1825.10**
**Minimum**	**4.03**	**22.33**	**396.49**	**902.96**	**0.95**	**10.25**	**124.13**	**166.51**
**Maximum**	**286.71**	**1210.36**	**14,022.68**	**38,132.49**	**71.55**	**865.38**	**9708.74**	**8988.98**

*Cmax* maximum concentration observed, *C0* initial concentration, *AUCall* total area under curve from 0-infinity, *AUMC* area under moment curve

#### Compartmental model diagnostics

The concentration–time curves for total and free ceftriaxone fit the population one-compartmental model. *Phoenix software collected data* demonstrates low values of Akaike information criterion (AIC) (191.35, 524.63) and Bayesian information criterion (BIC) (202.73, 536.01) for free and total ceftriaxone, respectively, in population one-compartmental model compared to those in population two-compartmental model (AIC = 200.85, 528.15 and BIC = 221.34, 548.64) Table 4.

**Table 4:** Compartmental model diagnostics

Source	AIC*	BIC**	RetCode ***	LogLik****	-2LL****	EpsShrinkage****
Population Model 1c Free Conc Multiplicative	191.3514	202.7348	3	− 90.6757	181.3514	0.29349
Population Model 2c Free Conc Multiplicative	200.8512	221.3412	3	− 91.4256	182.8512	0.33099
Population Model 1c Total Conc Multiplicative	524.6312	536.0145	2	− 257.316	514.6312	0.19803
Population Model 2c Total Conc Multiplicative	528.1524	548.6424	1	− 255.076	510.1524	0.17984

*AIC Akaike information criterion

**BIC Bayesian information criterion

***Retcode return code usually acceptable up to 3 Positive Retcode indicates the convergence of data

****LogLik,2LL,shrinkage, used in calculation of AIC

**Fig. 2:**
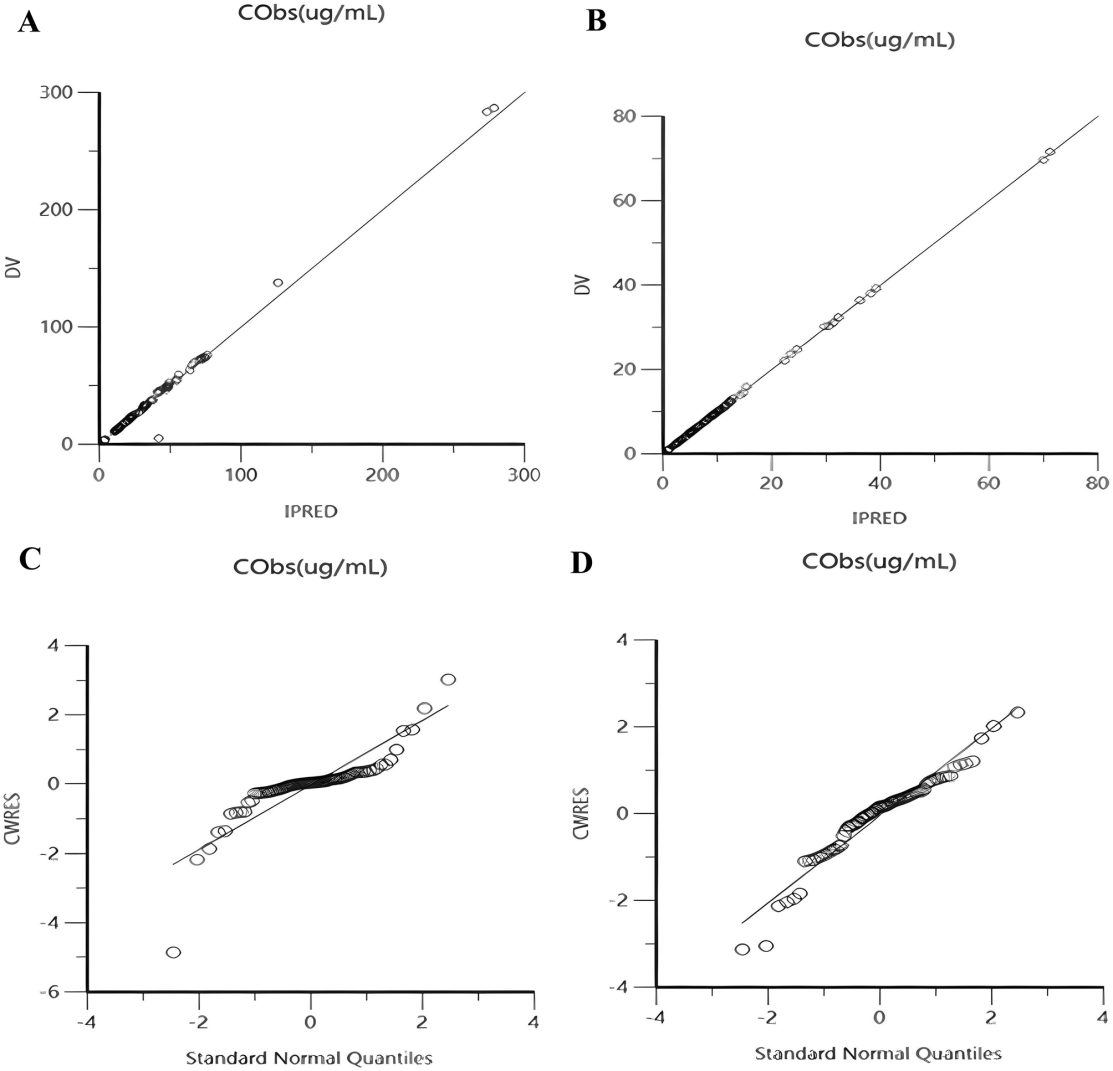
The excellent agreement between the observed concentrations and those predicted by the one-compartment model. (**A**) For total ceftriaxone; (**B**) for free ceftriaxone and plotting the residuals against standard normal quantiles. (**C**) For total ceftriaxone; (**D**) for free ceftriaxone

The excellent agreement between observed and predicted concentrations for population and individuals demonstrated that the one-compartment model could precisely represent the concentration–time courses of total and unbound ceftriaxone in 24 pediatric patients (Fig. 2A–B), (Figs. S3, S4, S5 and S6).

By plotting the residuals (difference between observed and predicted concentrations) against standard normal quantiles, the data fall very close to the diagonal line, meaning that the residuals are normally distributed (Fig. 2C–D).

#### Pharmacokinetic parameters results

Regarding the non-compartmental analysis results, all pharmacokinetic parameters obtained by phoenix software are illustrated in Table 2. Furthermore, exposure parameters including Cmax, Co, AUC AND AUCM are listed in Table 3.

#### Correlation between renal function and the free fraction of ceftriaxone

**Fig. 3:**
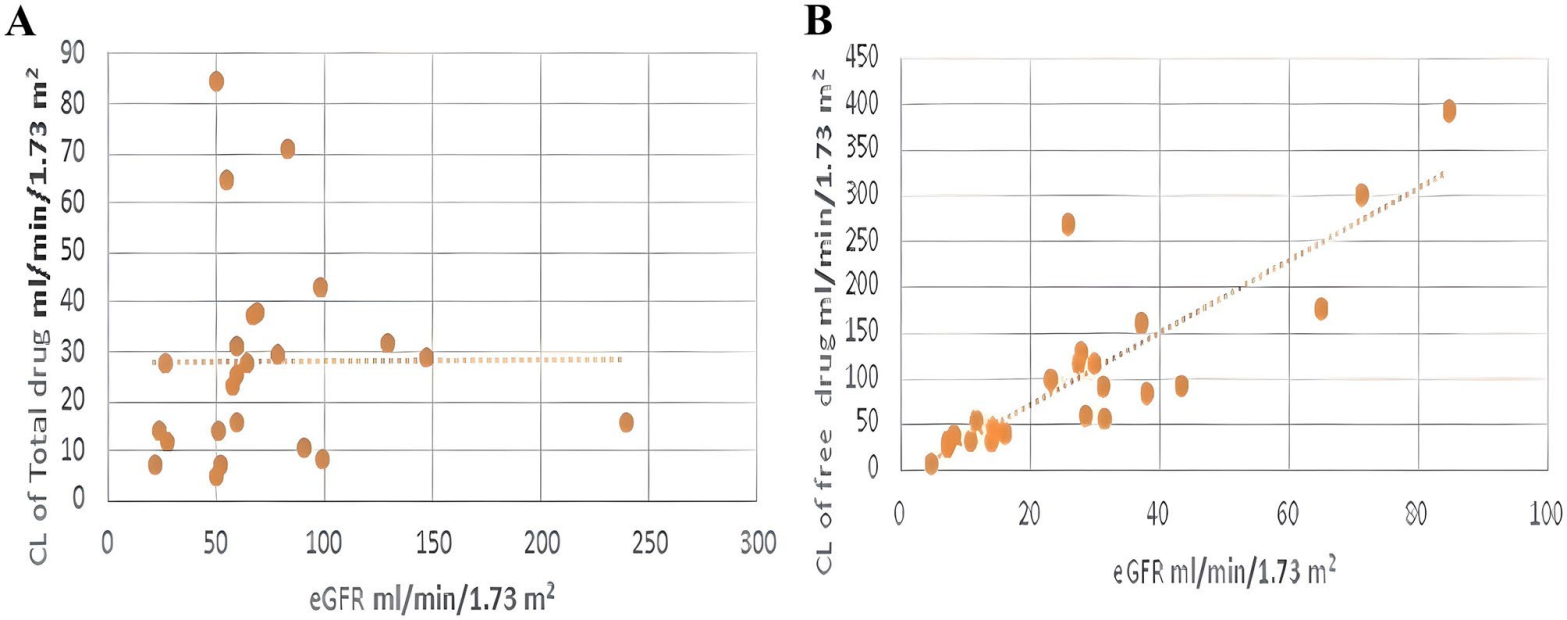
eGFR vs. Cl of free ceftriaxone in all patients. (**A**) For total ceftriaxone, *r*^2^ = 2 × 10–5; (**B**) for free ceftriaxone, *r*^2^ = 0.7252

The clearance of free ceftriaxone for all patients has a good correlation with the renal function of the patients(eGFR) [*r*^2^ = 0.7252, the regression equation that represents the clearance of the free ceftriaxone was Cl (ml/min/1.73 m^2^) = 3.9465 × eGFR(ml/min/1.73 m^2^) – 7.2258] while the clearance of total ceftriaxone for all patients has poor correlation with the renal function of the patients (eGFR) (*r*^2^ = 2 × 10^−5^) (Fig. 3).

## Discussion

Many analytical techniques determine the plasma concentration of ceftriaxone. One utilizes high-performance liquid chromatography with column Hypersil 250 × 4.6 mm and a mobile phase consisting of a mixture of tetra butyl ammonium hydroxide buffer and acetonitrile in a ratio of (70:30) [13]. Another study utilizes high-performance liquid chromatography with UV detection with octadecyl silica (ODS) column (Hydrosphere 150 × 4.6 mm, 5 μm) in an isocratic elution system with mobile phase A composed of methanol and 10 mM phosphoric acid (25:75, v/v) mixture solution and mobile phase B composed of methanol and water (80:20, v/v) mixture solution with detection at 280 nm [17]. Another study utilizes an ultra-high performance liquid chromatography with UV–visible detection C18 (2.1 × 100 mm; 1.8 μm) column protected by a VanGuard guard column. Both were placed into the column oven and maintained at 40 °C. The mobile phase was composed of a mixture of methanol and ammonium acetate 20 mM (21:79, v/v) with detection at 260 nm [14]. This study utilized this method with some modifications, as discussed in the Methods part.

In this observational study, we describe the pharmacokinetics of total and unbound ceftriaxone with clinical evaluation in 24 pediatric patients. Data for total and free ceftriaxone were best fit on a one-compartment model, based on lower values for both AIC and BIC values from one-compartment model compared to those from two-compartment model, with linear clearance of the free ceftriaxone. Some previous studies show data for free and total ceftriaxone fitted on a two-compartment model [18–20]. Other studies show data for ceftriaxone best fit on one compartment model, which agrees with our research [5].

As demonstrated in the results, all patients have a trough concentration of total ceftriaxone above the range of 0.5 to 2 ug/ml, which is adequate to achieve ceftriaxone’s target activity against most susceptible organisms. Patients with hypoalbuminemia (6 patients in our study) show an elevated free fraction up to 0.55 with a high volume of distribution of free ceftriaxone up to 30 L with a relatively long elimination half-life of about 10–15 h (except for patients with good kidney function show shorter elimination half-life of about 4 h and high drug clearance). These patients have eGFR of around 60 ml/min/1.73 m^2^ with relatively low clearance of total and free ceftriaxone attributed to a somewhat reduced kidney function (except for patients 3 and 4, in which the kidney function is perfect).

As in previous studies, not only bilirubin and albumin concentrations have an impact on the free fraction, but also the critical illness can do, as most of our patients were admitted to intensive care unit contributing to such elevated free fractions up to 0.6, which led to changes in the volume of distribution and clearance of the drug as discussed [9].

Furthermore, the critical illness has great impact on the augmented renal function supporting this the use of *Bedside Schwartz Formula* as mentioned at "Patients and Methods" section which consider the constant value to adjust the effect of critical illness to kidney function. In this study, the use of the Bedside Schwartz formula allowed for a more accurate estimation of renal function in critically ill patients and ensured that the impact of critical illness on drug clearance was appropriately accounted for. This is an important consideration in the management of critically ill patients, as accurate dosing of medications like ceftriaxone is crucial for achieving optimal therapeutic outcomes. This is obviously observed in patients 3, 4, 6, 13, 16 and 22 for whom the augmented renal function was clear [21].

Most adverse effects precipitated by ceftriaxone, especially in pediatrics, occur due to its inappropriate use [22]. Although none of the patients developed hyperbilirubinemia, the total bilirubin increased compared to baseline values. This increase is mild, but this can support the implication of ceftriaxone in cholestasis, the possibility of gallstone development, and the risk of ceftriaxone-induced cholestatic hepatitis. One of the adverse effects of ceftriaxone is cholestasis which can lead to biliary calculi and may lead to cholestatic hepatitis. Total bilirubin concentrations and liver enzymes, including ALT and AST, were mildly increased after treatment in these patients. The test which was performed is *T*-test from which changes were significant. After running exposure–response analysis there wasn’t any correlation observed suggesting that these changes are not concentration-dependent. A published case report showed a case of a 5-year-old boy presented with ceftriaxone-induced cholestatic hepatitis [23].

In the present study, no patients develop gallbladder stones after treatment but are still at risk. So, we warn the health care providers in our sitting that treatment with ceftriaxone requires monitoring for cholestasis and gallbladder stones to avoid this problem in patients. Around 50% of our patients have impaired renal function, which contributes to reduced protein binding by unknown mechanisms. This may be due to waste products accumulated in the body that may displace ceftriaxone from its binding sites.

In many previous studies, the drug’s protein binding changes have little clinical importance. This can be illustrated by a condition that causes an acute increase in the free fraction causing the excess of the drug to be eliminated keeping a steady fraction of the drug. But this depends on kidney function, and in such patients with impaired kidney function, accumulation of ceftriaxone occurs, leading to undesirable effects of the drug. One has been reported as discussed, so dosage adjustment was necessary [24].

Regarding drug-drug interaction screening in our patients, only two pharmacodynamic interactions have been reported in patient number 1. One of them is serious between ceftriaxone and enoxaparin, leading to increased prothrombin activity as a synergistic effect. The second pharmacodynamic interaction is minor between ceftriaxone and furosemide, leading to additive nephrotoxicity. From these interactions, the second one added a negative effect on renal function as observed (eGFR < 30 ml/min/1.73 m^2^) also affecting the clearance of ceftriaxone (reduced CL = 412.42 ml/h).

The covariate analysis was already done, and found that only body weight has a significant effect as covariate (***p*** = 015,*** r2*** = 0.4) on clearance of ceftriaxone in all patients together with. But, on the other hand, we conduct this study on different ages with different clinical indications for ceftriaxone and administering different doses ranging from 50 to 100 mg/kg in order to evaluate the PK of ceftriaxone individually and evaluate the cases clinically with possible drug-drug interaction or another associated medical conditions. This could be appropriate for future population PK of ceftriaxone focusing on one specific clinical indication (as pneumonia, or meningitis) with fixed doses to run a such covariate analysis.

Limitations of this study involve a small sample size, although determined by IRB, and this is attributed to a small number of patients admitted to the hospital who fit the inclusion criteria; a large-scale clinical trial is required in different areas worldwide utilizing more advanced techniques for protein binding study which is not possible for us due to financial issues. Also, regarding sampling time, we faced such a problem due to ethical issues only specific time during the day, which is fixed according to hospital regulations without the chance of obtaining samples at different time intervals from the last dose.

## Conclusion

Data for total and free ceftriaxone were best fit on a one-compartment model with linear clearance of the free ceftriaxone. The current dosing regimen of ceftriaxone (50 to 100 mg/kg) provides appropriate pathogen exposure in most critically ill pediatric patients. Total bilirubin concentration was within the normal range, and an increase in total bilirubin after treatment with ceftriaxone was observed. Also, liver enzymes, including ALT and AST, are mildly increased. So, liver function tests and total bilirubin monitoring are necessary during treatment with ceftriaxone, especially in pediatric patients and those administered the ceftriaxone for a long duration, more than 5 days, or use another agent in patients with high baseline values to avoid the development of cholestasis. Also, large-scale multi-center pharmacokinetic studies involving different ethnicity from the pediatric population are recommended to report various variabilities in ceftriaxone pharmacokinetics which affect its therapeutic outcomes and the possibility of the emergence of its undesirable effects.

### Supplementary Information

Below is the link to the electronic supplementary material.

Supplementary file1 (DOCX 761 KB)

Supplementary file2 (PDF 495 KB)

## Data Availability

Data supporting results in this study including chromatograms generated by HPLC apparatus, data for direct and total bilirubin, associated clinical conditions & drug-drug interactions, HPLC Method Validation results, individual plots for observed and predicted concentrations of ceftriaxone generated by Phoenix Winnonlin Program software were included in the Supplementary file. Other data required will be available on request from the corresponding author.

## Abbreviations

95% CI: 95% Confidence interval
AIC: Akaike information criterion
ALT: Alanine aminotransferase
AST: Aspartate aminotransferase
AUCall: Total area under curve from 0-infinity
AUMC: Area under moment curve.
BLOQ: Below the limit of quantification
BIC: Bayesian information criterion
C0: Initial concentration
CL: Clearance
Cmax: Maximum concentration observed
eGFR: Estimated glomerular filtration rate
EIPICO: Egyptian International Pharmaceutical Industries Company
EUCAST: European Committee on antimicrobial susceptibility testing
FDA: Food and Drug Administration
fT: Free fraction of the drug
HPLC: High-performance liquid chromatography
IRB: Institutional Review Board
LLOQ: Lower limit of quantification
LOD: Limit of detection
LOQ: Limit of quantification
MIC: Minimum inhibitory concentration
mM: Millimole
NCA: Non compartmental analysis
PBPs: Penicillin-binding proteins
RSD%: Relative standard deviation
S: The slope
Scr: Serum creatinine
SD: Standard deviation
Vss: The volume of distribution at steady state

## Footnotes

1 Doses were determined by the health care provider according to the actual body weight.
