# Supplementary material for: Evaluation of ceftriaxone pharmacokinetics in hospitalized Egyptian pediatric patients
Source: Eur J Pediatr. 2023 Jul 24;182(10):4407–20. doi: 10.1007/s00431-023-05091-0 (PMC10587312; doi:10.1007/s00431-023-05091-0)

Supplementary Figures

Fig. S1A


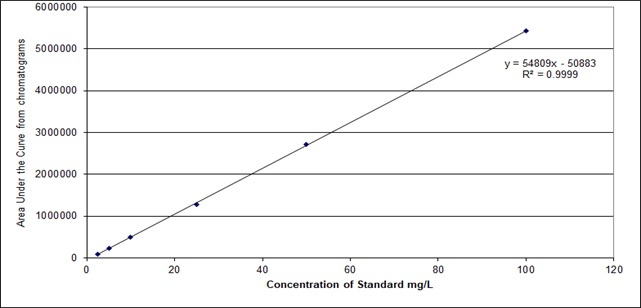


Fig. S1B


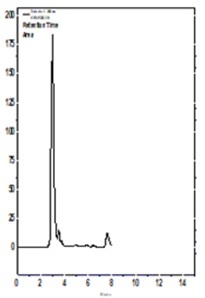


Fig. S1C


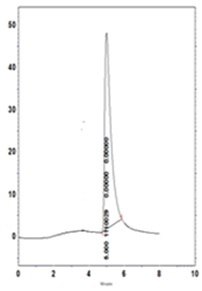


Fig. S1D


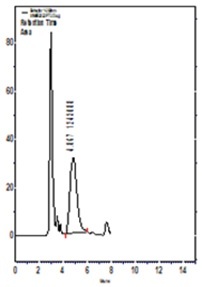


Fig. S2A


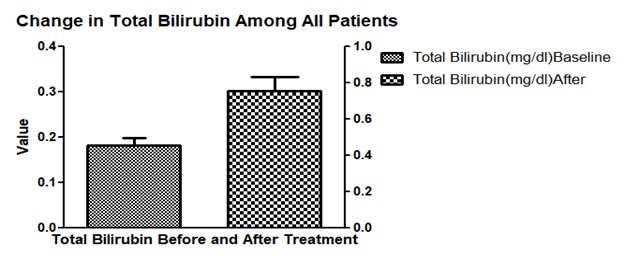


Fig. S2B


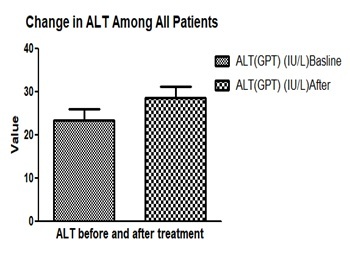


Fig. S2C


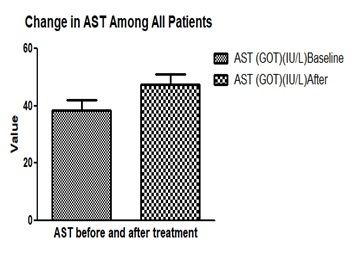


Fig. S3-1


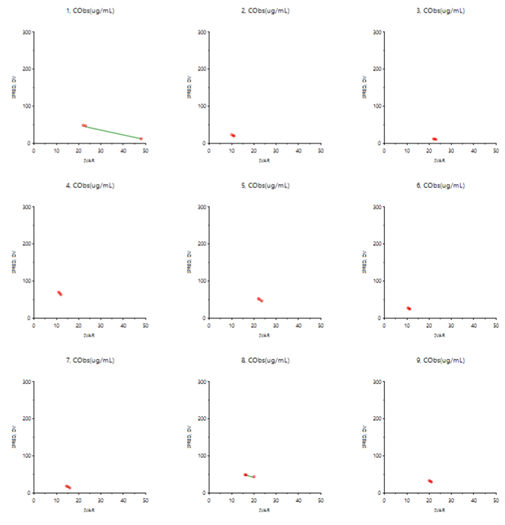


Fig. S3-2


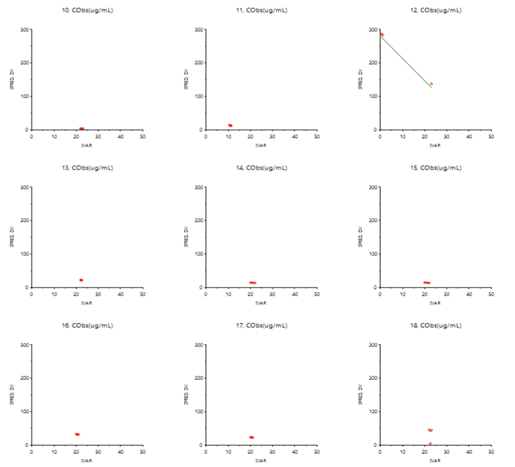


Fig. S4


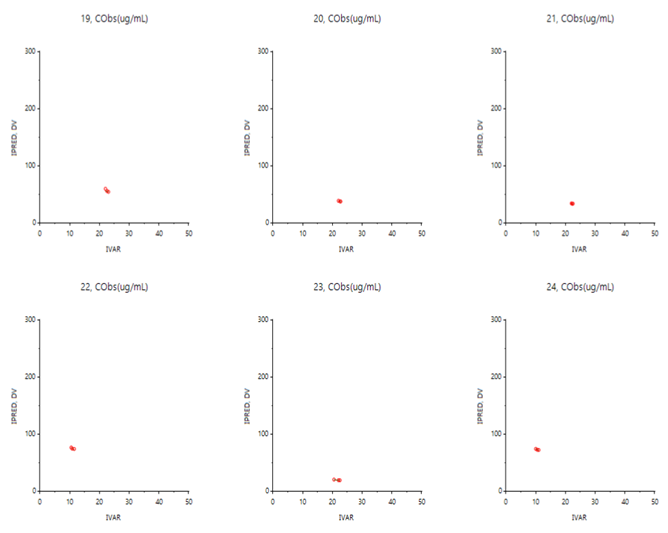


Fig. S5-1


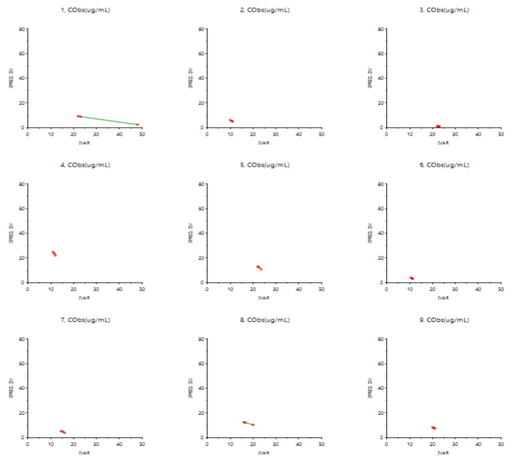


Fig. S5-2


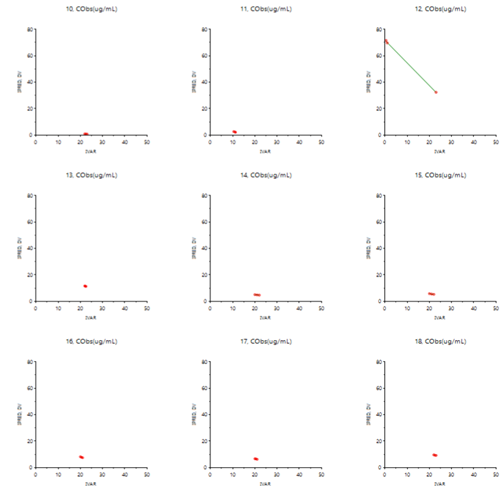


Fig. S6


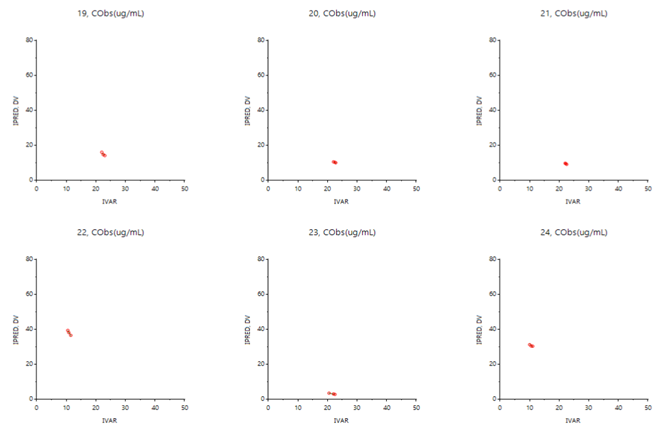


Fig. S7-1


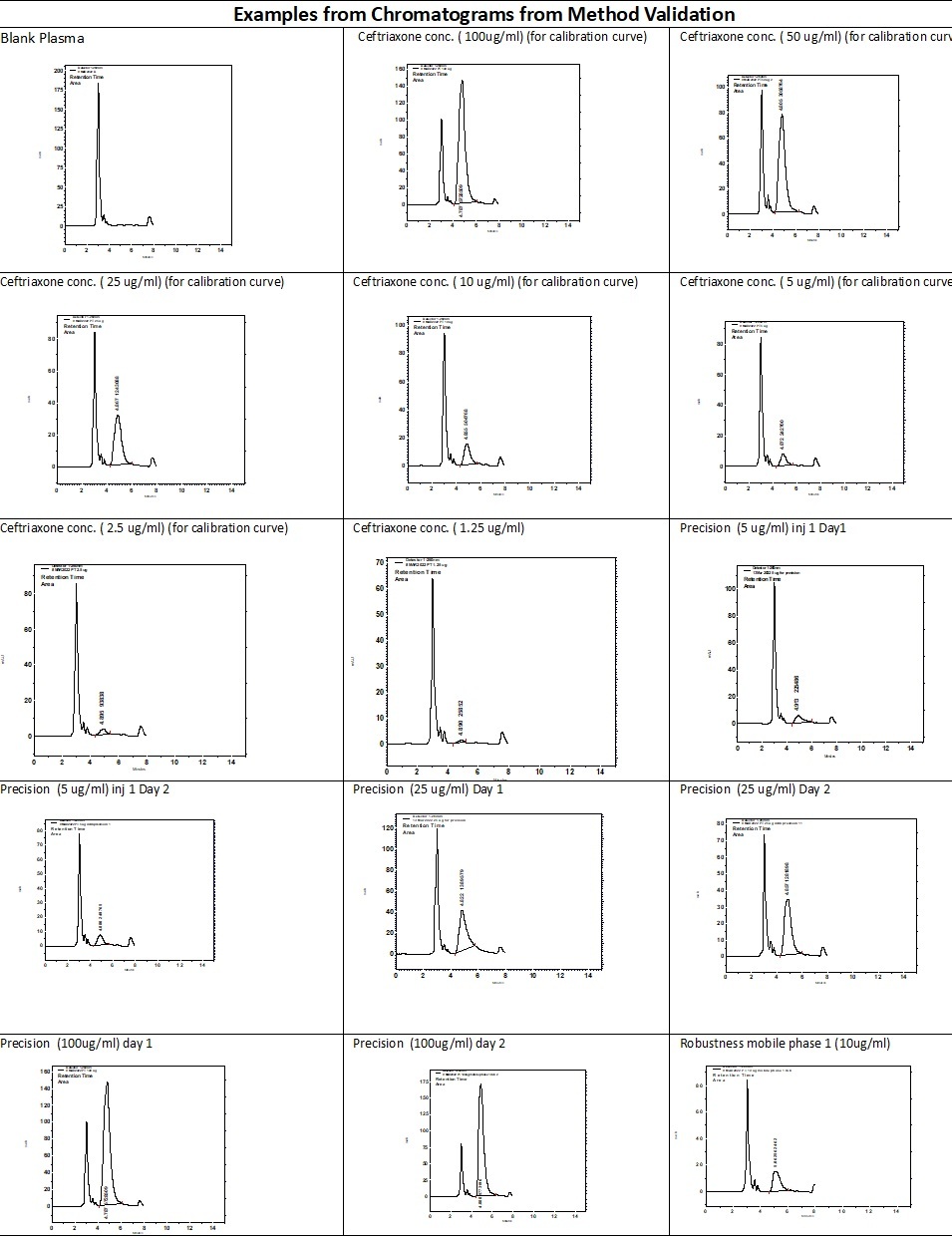


Fig. S7-2


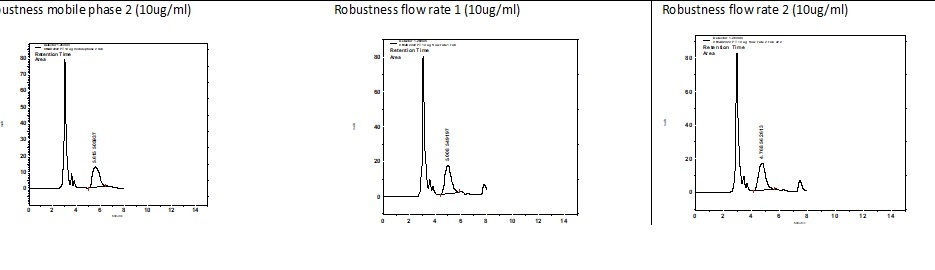


Fig. S8-1


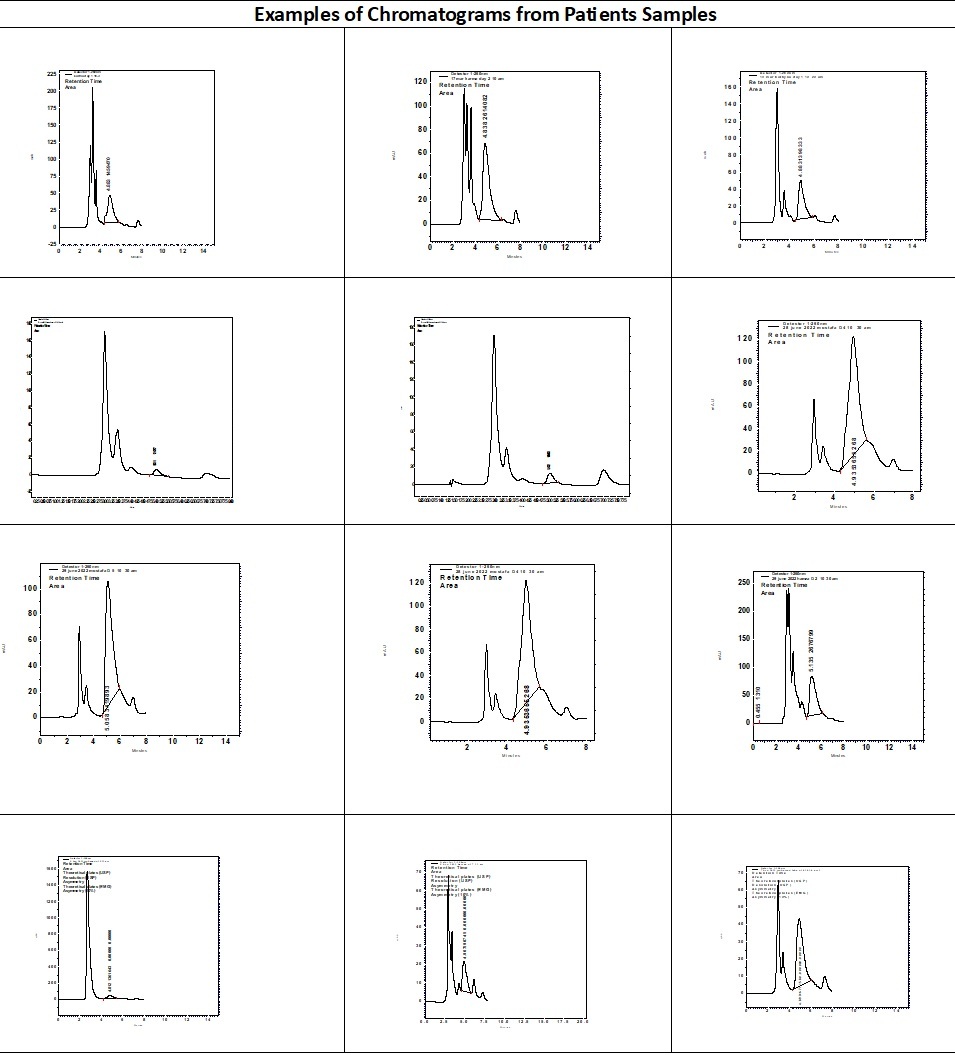


Fig. S8-2


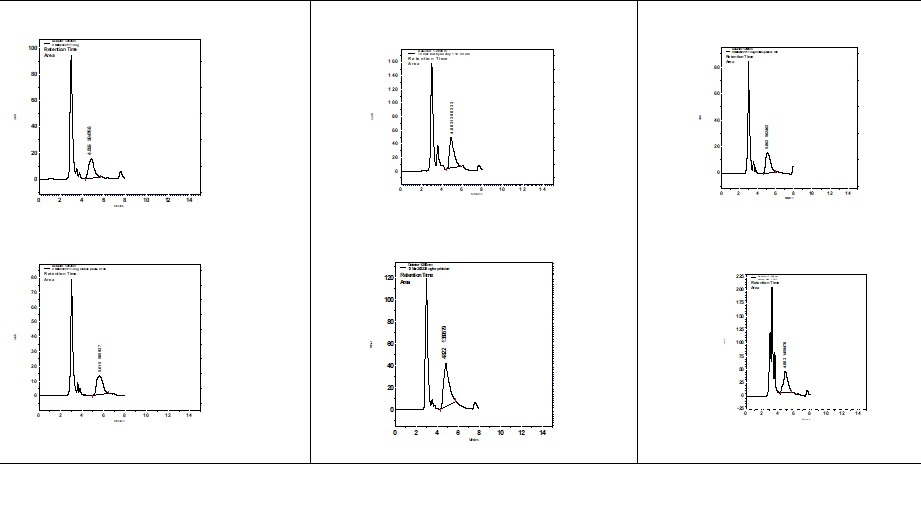

Supplement: Supplementary file 1 — Supplementary file1 (DOCX 761 KB) [file 431_2023_5091_MOESM1_ESM.docx]
