# Supplementary material for: Evaluation of ceftriaxone pharmacokinetics in hospitalized Egyptian pediatric patients
Source: Eur J Pediatr. 2023 Jul 24;182(10):4407–20. doi: 10.1007/s00431-023-05091-0 (PMC10587312; doi:10.1007/s00431-023-05091-0)
Supplement: Supplementary file 2 — Supplementary file2 (PDF 495 KB) [file 431_2023_5091_MOESM2_ESM.pdf]

List of Supplement Tables	
**Table**	**Title**
**Table S1**	**Values for Changing in total and direct bilirubin**
**Table S2**	**Validation parameters for the developed HPLC method for determination of Ceftriaxone in plasma**
**Table S3**	**Robustness of HPLC method for determination of Ceftriaxone in plasma**
**Table S4**	**Associated medical conditions and drug-drug interactions observed in patients**

Table S1 : Values for Changing in total and direct bilirubin

Patient's No.	Total		Direct		% Direct	
	**B**	**A**	**B**	**A**	**B**	**A**
**1**	0.2	0.28	0.1	0.21	**50.00%**	**75.00%**
**2**	0.2	0.25	0.08	0.175	**40.00%**	**70.00%**
**3**	0.1	0.3	0.052	0.219	**52.00%**	**73.00%**
**4**	0.08	0.2	0.0424	0.16	**53.00%**	**80.00%**
**5**	0.3	0.4	0.138	0.28	**46.00%**	**70.00%**
**6**	0.1	0.2	0.035	0.152	**35.00%**	**76.00%**
**7**	0.2	0.27	0.092	0.1944	**46.00%**	**72.00%**
**8**	0.1	0.2	0.042	0.154	**42.00%**	**77.00%**
**9**	0.1	0.23	0.049	0.1518	**49.00%**	**66.00%**
**10**	0.2	0.25	0.088	0.1775	**44.00%**	**71.00%**
**11**	0.2	0.29	0.078	0.2175	**39.00%**	**75.00%**
**12**	0.1	0.22	0.035	0.1628	**35.00%**	**74.00%**
**13**	0.4	0.9	0.144	0.711	**36.00%**	**79.00%**
**14**	0.1	0.2	0.039	0.164	**39.00%**	**82.00%**
**15**	0.2	0.25	0.094	0.1875	**47.00%**	**75.00%**
**16**	0.2	0.27	0.064	0.2025	**32.00%**	**75.00%**
**17**	0.1	0.19	0.046	0.1235	**46.00%**	**65.00%**
**18**	0.1	0.15	0.05	0.1125	**50.00%**	**75.00%**
**19**	0.2	0.27	0.092	0.2025	**46.00%**	**75.00%**
**20**	0.28	0.35	0.126	0.2625	**45.00%**	**75.00%**
**21**	0.2	0.4	0.092	0.336	**46.00%**	**84.00%**
**22**	0.29	0.5	0.1247	0.375	**43.00%**	**75.00%**
**23**	0.2	0.4	0.082	0.276	**41.00%**	**69.00%**
**24**	0.2	0.27	0.086	0.2052	**43.00%**	**76.00%**
**Minimum**	**0.08**	**0.15**	**0.035**	**0.1125**	**0.32**	**0.65**
**Maximum**	**0.4**	**0.9**	**0.144**	**0.711**	**0.53**	**0.84**
**Median**	**0.2**	**0.27**	**0.081**	**0.19845**	**0.445**	**0.75**

Table S2 Validation parameters for the developed HPLC method for determination of Ceftriaxone in plasma

Parameter	Value
**Wavelength**	260 nm
**Linear range**	2.5 – 100.0 μg mL^{-1}
**LOD (μg ml^{-1})**	0.738
**LOQ (μg ml^{-1})**	2.238
**Regression equation:**	Ceftriaxone Concentration = 54809 x Peak Area - 50883
**Slope**	54809
**Intercept**	-50883
**Correlation coefficient (r^2)**	0.9999
**Accuracy(%recovery) ± SD**	100.79 ± 2.10
**RSD (%)**	2.11
**System suitability test**	
**Retention time**	5.00
**Number of theoretical plates (N)**	1125
**Height equivalent to theoretical plates (HETP)**	0.22
**Tailing factor**	1.33
**Resolution (Rs)**	4.26
**Intra-day Precision (Recovery % ± RSD%)**	
**5 µg mL^{-1}**	107.62 ± 2.03
**25 µg mL^{-1}**	95.64 ± 1.39
**100 µg mL^{-1}**	99.41 ± 1.23
**Inter-day Precision (Recovery % ± RSD%)**	
**5 µg mL^{-1}**	104.35 ± 4.10
**25 µg mL^{-1}**	99.82 ± 3.37
**100 µg mL^{-1}**	98.99 ± 1.41

HPLC; High-performance liquid chromatography ,**LOD;** limit of detection, **LOQ;** limit of quantification, **SD;** standard deviation, **RSD%**; Relative standard deviation

Table S3 Robustness of HPLC method for determination of Ceftriaxone in plasma

Parameter	Methanol (%) change				Flow rate(mL/min) change			
	79	80	81	RSD%	0.98	1.00	1.02	RSD%
**Retention time**	4.855	5.062	5.215	3.58	5.008	4.855	4.768	2.49
**Peak Area**	504768	522462	505937	1.94	529197	504768	522613	2.44

RSD% = Relative standard deviation

**Table S4 :** Associated medical conditions and drug-drug interactions observed in patients			
**Item**		**Count (Percent if present**	**Notes**
**Associated Medical Conditions (7 conditions have been found)**	**a. Haemolytic Uremic Syndrome (HUS)**	**1 (≈ 4.167 %)**	Patient **1**
	**b. Bronchopulmonary Dysplasia**	**1 (≈ 4.167 %)**	Patient **2**
	**c. Nephrotic Syndrome**	**2 (≈ 8.33 %)**	Patients **4 & 18**
	**d. Nephrotic Syndrome + Hypercholesterolemia**	**1 (≈ 4.167 %)**	Patient **3**
	**e. Chronic Renal Failure**	**1 (≈ 4.167 %)**	Patient **5**
	**f. Convulsions**	**3 (12.5 %)**	Patients **7, 9 & 10**
	**g. Dehydration**	**2 (≈ 8.33 %)**	Patients **14 & 15**
**Drug-Drug interactions detected (12 interactions detected; 6 pharmacodynamic & 6 pharmacokinetic)**	**a. Ceftriaxone + Enoxaparin**	**1 (≈ 4.167 %)**	Patient **1** (**Serious interaction:** ceftriaxone ↑ activity of prothrombin leading to synergistic effect) **PD**
	**b. Ceftriaxone + Furosemide**	**1 (≈ 4.167 %)**	Patient **1** (**Minor interaction:** ↑ risk of nephrotoxicity) **PD**
	**c. Ceftazidime + Furosemide**	**1 (≈ 4.167 %)**	PT **1** (**Minor interaction:** ↑ risk of nephrotoxicity) **PD**
	**d. Paracetamol + Enoxaparin**	**1 (≈ 4.167 %)**	Patient **1** (**Minor interaction:** paracetamol ↑ activity of enoxaparin) **PD**
	**e. Paracetamol + Metronidazole**	**4 (≈ 16.67 %)**	Patient**s 3, 4, 13 & 23**(**Minor interaction: Metronidazole** ↑ level of Paracetamol) **PK**
	**f. Methylprednisolone + Metronidazole**	**1 (≈ 4.167 %)**	Patient **4** (**Minor interaction:** Metronidazole ↑ level of Methylprednisolone) **PK**
	**g. Omeprazole + Fluconazole**	**1 (≈ 4.167 %)**	Patient **5** (**Minor interaction**: Fluconazole ↑ level of Omeprazole) **PK**
	**h. Ciprofloxacin + Dexamethasone**	**1 (≈ 4.167 %)**	Patient **6** (**Minor interaction**: ↑ risk of tendon rapture) **PD**
	**i. Paracetamol + Valproic acid**	**2 (≈ 8.33 %)**	Patient **7& 16** (**Minor interaction**: Valproic acid ↓ level of Paracetamol) **PK**
	**j. Midazolam + Dexamethasone**	**2 (≈ 8.33 %)**	Patients **9 & 12** (**Minor interaction**: Dexamethasone ↓ level of Midazolam) **PK**
	**k. Paracetamol + Levetiracetam**	**3 (12.5 %)**	Patients **10, 12 & 16** (**Minor interaction**: Levetiracetam ↓ level of Paracetamol) **PK**

PD = Pharmacodynamic interaction **, PK=** Pharmacokinetic Interaction

List of Supplement Figures	
**Figure**	**Caption**
**Figure S1**	Calibration Curve of Standard Ceftriaxone in Plasma AND HPLC Chromatograms of Blank plasma, Standard Ceftriaxone, and Standard Ceftriaxone in plasma. **A:** Calibration Curve of Standard Ceftriaxone in Plasma SD = 2.11; **B:** HPLC Chromatogram of Blank Plasma; **C:** HPLC Chromatogram of Standard Ceftriaxone ; **D:** HPLC Chromatogram of Standard Ceftriaxone in Plasma
**Figure S2**	Changes in total bilirubin and liver enzymes of 24 pediatric patients during treatment with ceftriaxone. **A:** represents changes in Total Bilirubin, p-value < 0.0001, 95% CI from -0.1607 to -0.08017 and r = 0.8349; **B**: represents changes in Alanine Aminotransferase, p-values < 0.0001, 95% CI values from -6.409 to -4.225 and r = 0.9802; **C**: represents changes in Aspartate Aminotransferase, p-values < 0.0001, 95% CI values from -12.55 to -5.144 and r = 0.8678
**Figure S3**	Observes versus Predicted Total Ceftriaxone concentrations from patient 1 to patient 18
**Figure S4**	Observes versus Predicted Total Ceftriaxone concentrations from patient 19 to patient 24
**Figure S5**	Observes versus Predicted Free Ceftriaxone concentrations from patient 1 to patient 18
**Figure S6**	Observes versus Predicted Free Ceftriaxone concentrations from patient 19 to patient 24
**Figure S7**	Examples of Chromatograms from HPLC for Method Validation
**Figure S8**	Examples of Chromatograms from HPLC for Patients' Samples

A

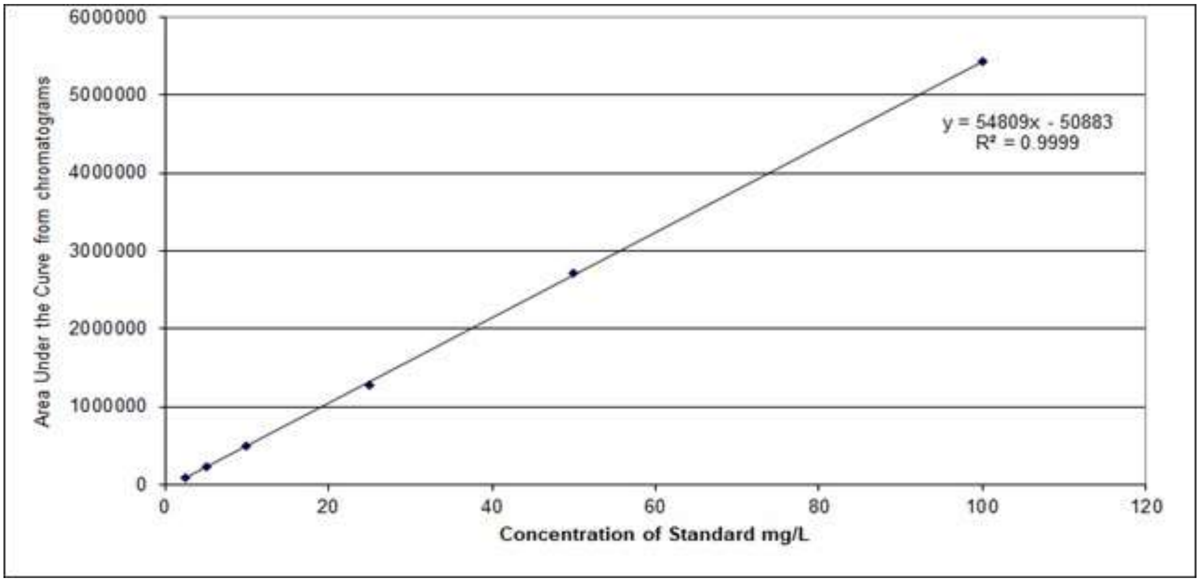

B

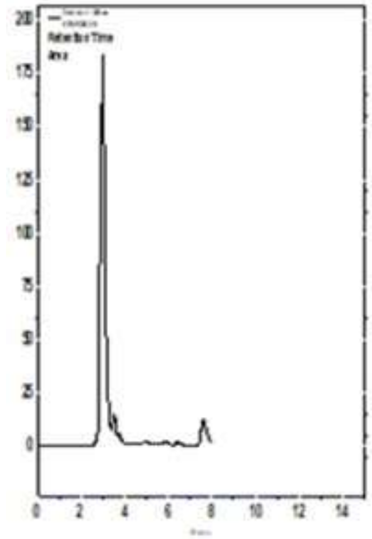

C

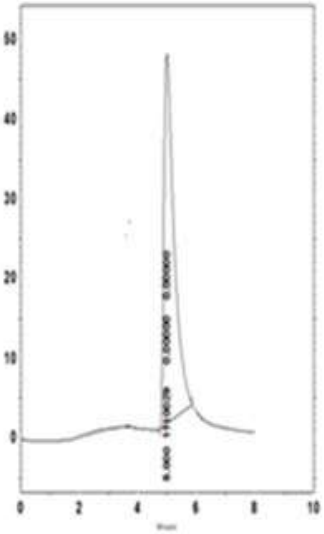

D

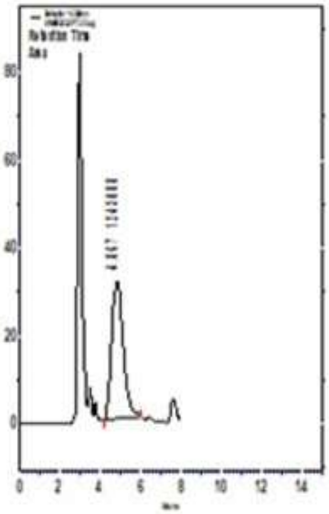

Figure S1 Calibration Curve of Standard Ceftriaxone in Plasma AND HPLC Chromatograms of Blank plasma, Standard Ceftriaxone, and Standard Ceftriaxone in plasma. A: Calibration Curve of Standard Ceftriaxone in Plasma SD = 2.11; B: HPLC Chromatogram of Blank Plasma; C: HPLC Chromatogram of Standard Ceftriaxone ; D: HPLC Chromatogram of Standard Ceftriaxone in Plasma

A

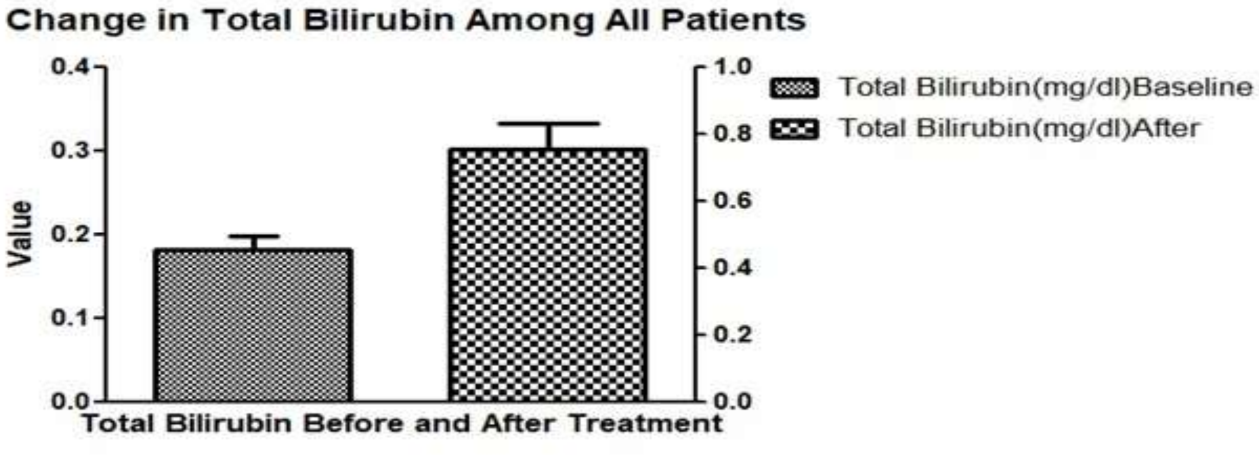

B

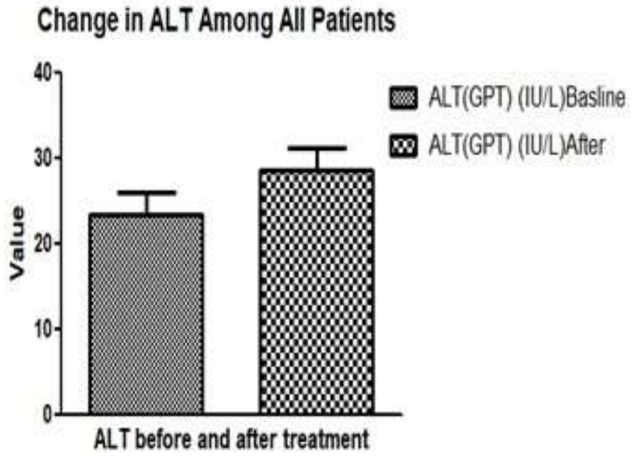

C

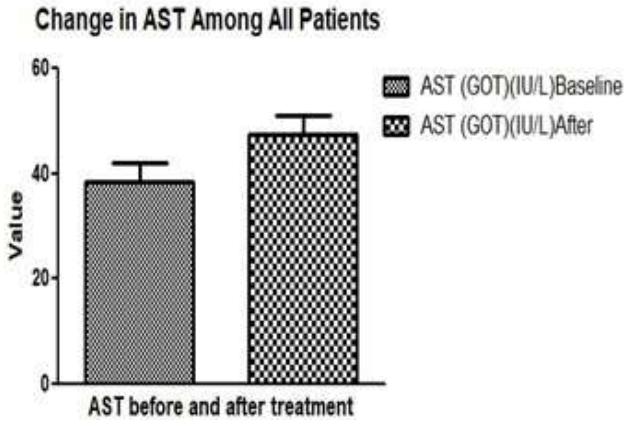

Figure S2 Changes in total bilirubin and liver enzymes of 24 pediatric patients during treatment with ceftriaxone. **A:** represents changes in Total Bilirubin, *p-value* < 0.0001, *95% CI from -0.1607 to -0.08017* and $r = 0.8349$; **B**: represents changes in Alanine Aminotransferase, *p-values* < 0.0001, *95% CI values from -6.409 to -4.225* and $r = 0.9802$; **C**: represents changes in Aspartate Aminotransferase, *p-values* < 0.0001, *95% CI values from -12.55 to -5.144 and* $r = 0.8678$

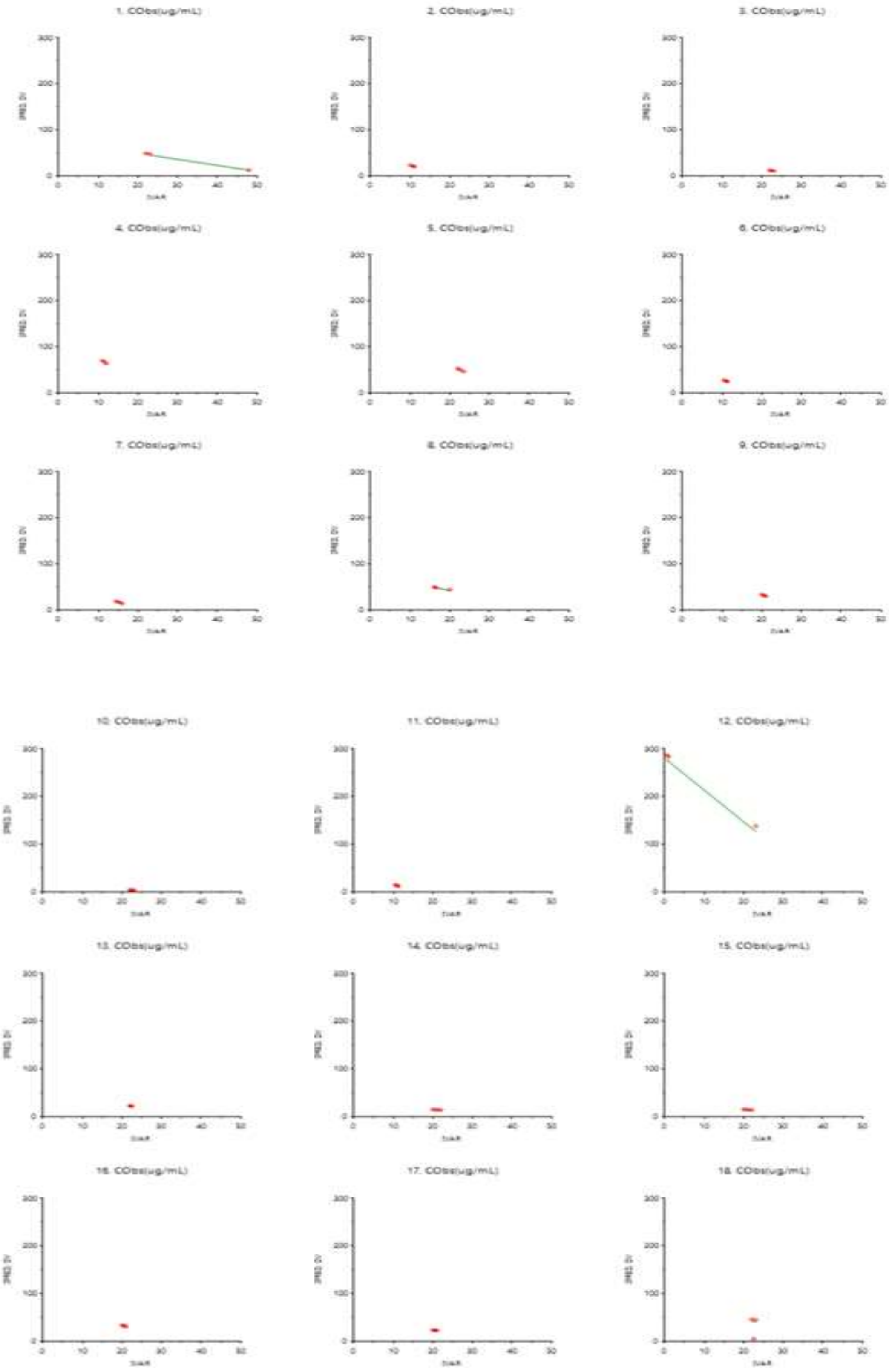

Figure S3 Observes versus Predicted Total Ceftriaxone concentrations from patient 1 to patient 18

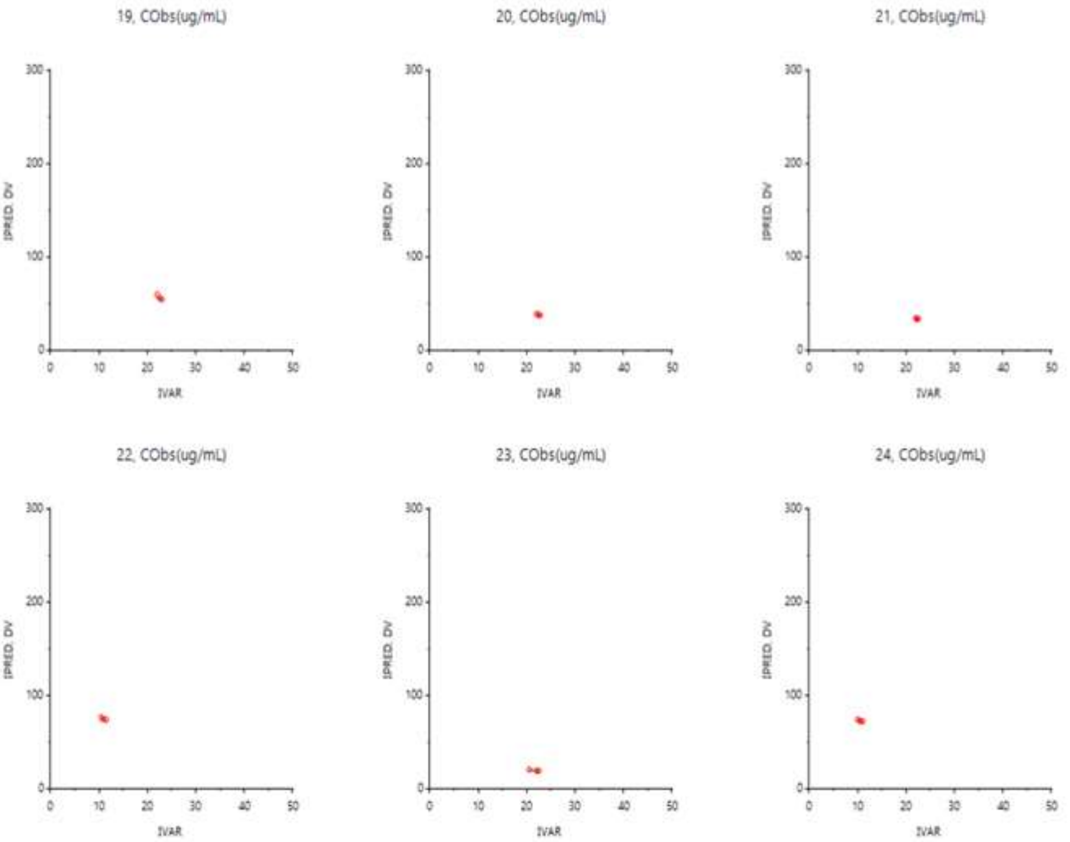

Figure S4 Observes versus Predicted Total Ceftriaxone concentrations from patient 19 to patient 24

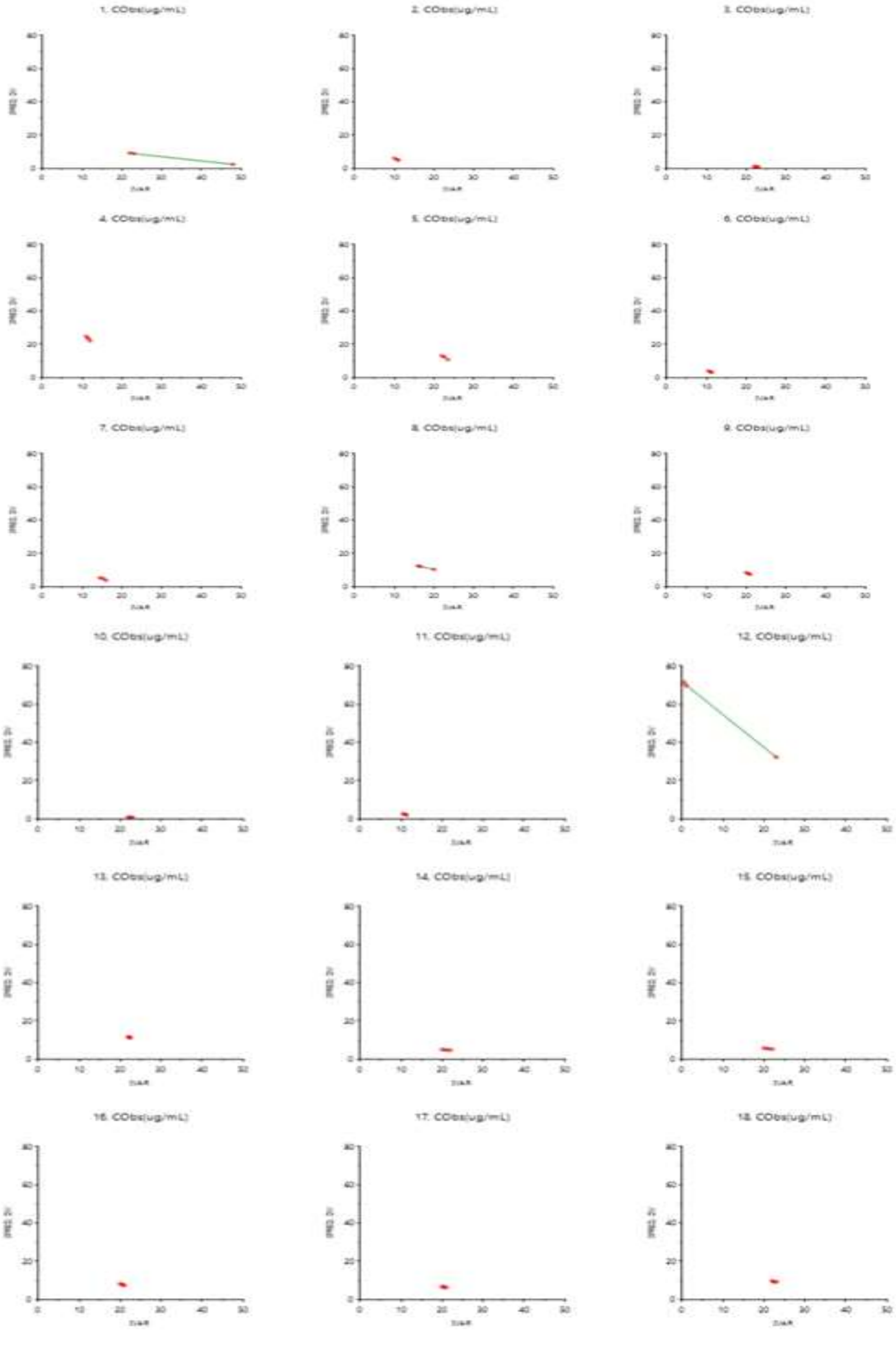

Figure S5 Observes versus Predicted Free Ceftriaxone concentrations from patient 1 to patient 18

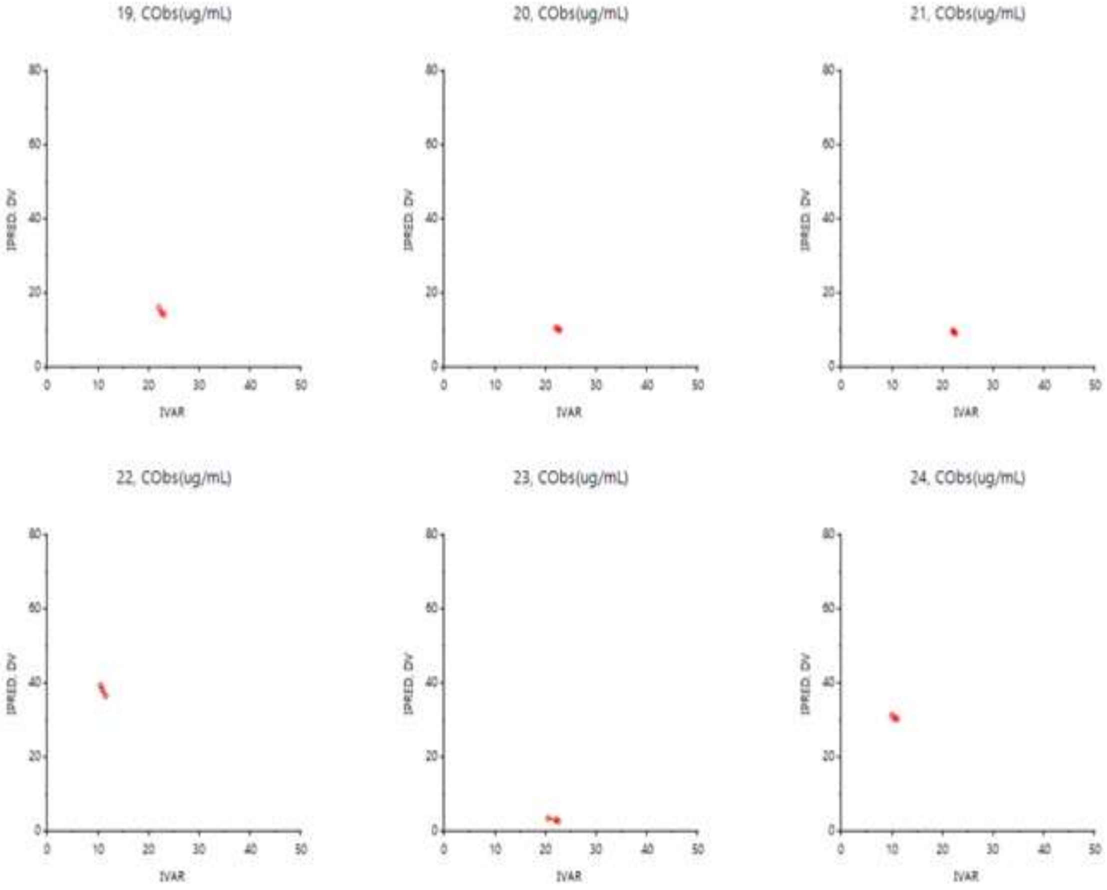

Figure S6 Observes versus Predicted Free Ceftriaxone concentrations from patient 19 to patient 24

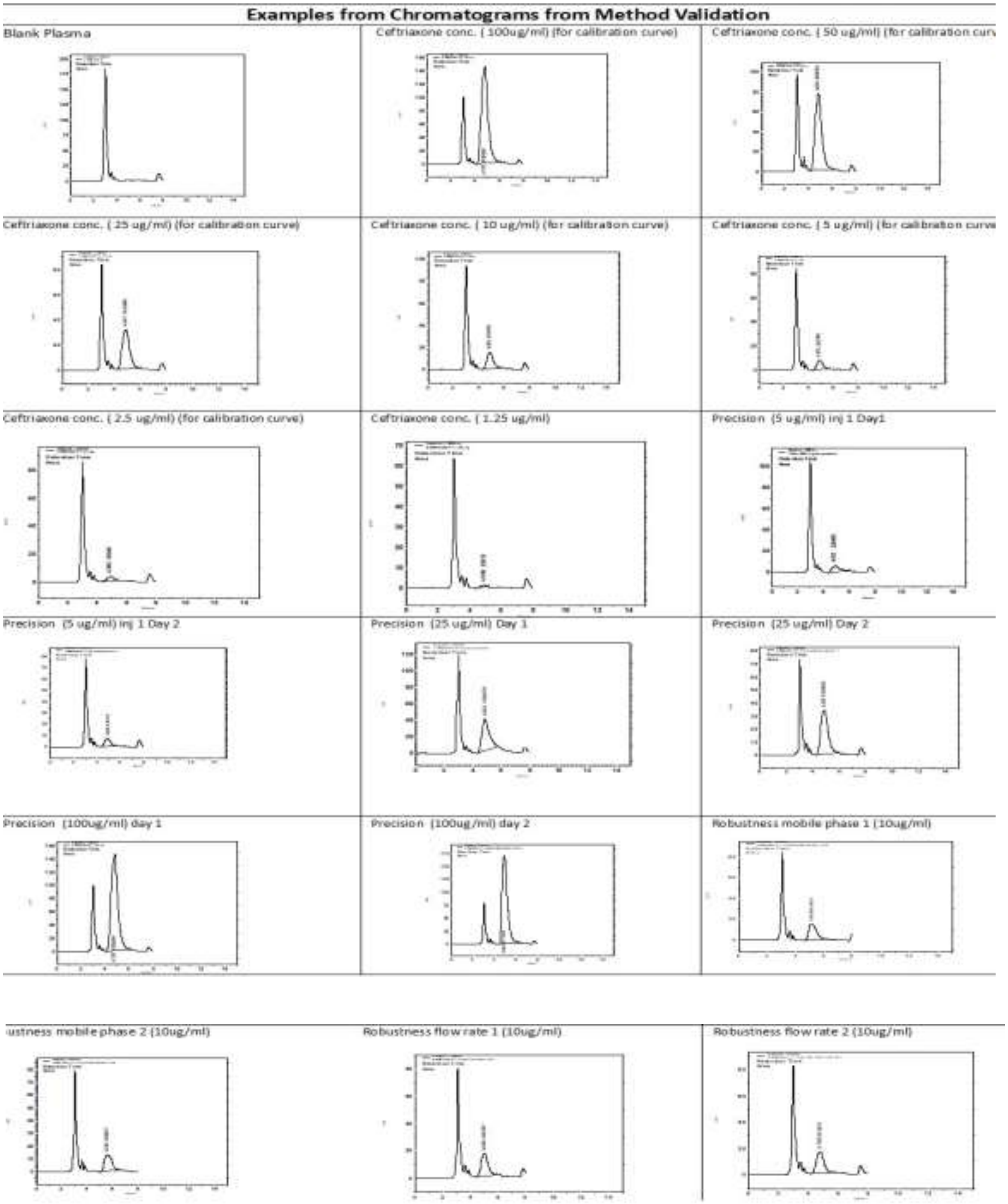

Figure S7 Examples of Chromatograms from HPLC for Method Validation

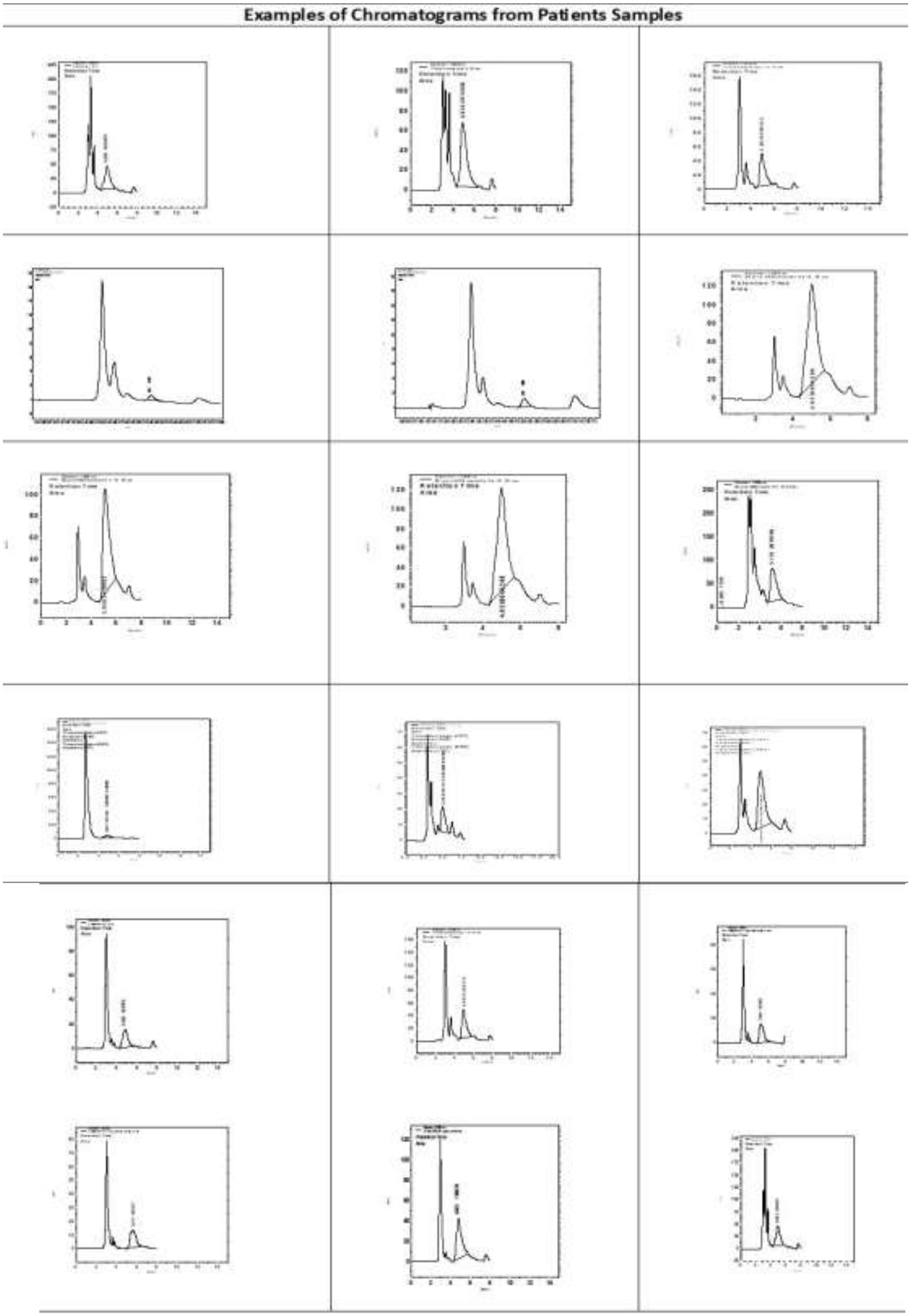

Figure S8 Examples of Chromatograms from HPLC for Patients' Samples

Operational Design of the Study

1. Study design: Cross-section study.

2. Steps of performance:

2.1. Patients and Clinical Samples

This pharmacokinetic study was conducted from May 2020 to November 2020 to assess the pharmacokinetics of total and unbound Ceftriaxone in Egyptian Pediatric Patients.

First, the characteristics and laboratory data of the patients included in this study are obtained from patients' files at baseline and after completion of treatment at Zagazig university pediatric hospital. Then, suitable doses of Ceftriaxone were administered to each patient. Blood samples were obtained opportunistically among available specimens to capture one or two dosing intervals, with three samples from each patient, which were then stored immediately after collection at 2-8 $_{o}$C in the Icebox and after that, plasma obtained then analyzed using HPLC method.

2.2. Experimental Analysis of Ceftriaxone Using HPLC Technique

2.2.1. Apparatus and Software:

Analysis was carried out on Thermo Fisher Scientific® HPLC system composed of binary pumps, autosampler with loop size was 10 µl, Photodiode array detector (PDA) with detection at 260 nm and Chromquest 5.0 software (Thermo Electron Corp., Bellefonte, PA, USA).

2.2.2. Materials and Reagents:

Ceftriaxone analytical standard was kindly supplied from Epico®. Methanol, acetonitrile and ammonium acetate (HPLC grades) were purchased from Fisher Scientific, USA. Double distilled water was applied all over the experiments and prepared in-house.

2.2.3. Standard Solutions:

A stock solution of ceftriaxone was prepared by dissolving 20 mg of pure drug in 100 mL double distilled water to get a stock solution with a concentration of 200 µg mL-1.

2.2.4. Chromatographic conditions

The conditions used have some modifications:

Samples were analyzed by HPLC using Agenla Technologies C18 column (250 mm×4.6mm, 5µm particle size) maintained at 25ºC temperature. Elution pumps ran an isocratic flow using a mobile phase consisting of methanol and ammonium acetate 20 mM buffer solution (0.77 g in 500 ml distilled water) in the ratio of (79%: 21% v/v) at 1 ml/min flow rate. The autosampler utilizes water as a rinse solution, and the injection volume was 10 µl. Detection was at 260 nm with a run time of 8 minutes.

2.2.5. Sample pre-treatment

Blood samples were collected from patients (when available,2 ml) in heparin tubes and then centrifuged to obtain plasma. 250 µl of plasma is mixed with 250 µl of cold acetonitrile in an Eppendorf tube and then centrifuged at 8000 rpm for 6 min at 4 ˚C. The supernatant is then filtered and injected into the HPLC system for analysis.

2.3. Pharmacokinetic Analysis

The pharmacokinetics of total and free ceftriaxone were analyzed separately with good fitting on a one-compartment model using Phoenix Winnonlin Program® (8.3.5.340, Core version 06Feb2020). Then the pharmacokinetic parameters were obtained and used to simulate the plasma concentration-time curve for both total and free ceftriaxone for each patient.

2.4. Statistical Analysis

Prism software was used for statistical analysis of the changed laboratory values.

2.5. Ethical Approval

The ethics committee represented by the Institutional Review Board (IRB) at the Faculty of Medicine, Zagazig University, approved the process of collecting blood samples from pediatric patients opportunistically during the duration of treatment (Approval Number; ZU-IRB#6070/26/4/2020).
